# A New Set of ESTs from Chickpea (*Cicer arietinum* L.) Embryo Reveals Two Novel F-Box Genes, *CarF-box_PP2* and *CarF-box_LysM*, with Potential Roles in Seed Development

**DOI:** 10.1371/journal.pone.0121100

**Published:** 2015-03-24

**Authors:** Shefali Gupta, Vanika Garg, Sabhyata Bhatia

**Affiliations:** National Institute of Plant Genome Research, New Delhi, India; National Key Laboratory of Crop Genetic Improvement, CHINA

## Abstract

Considering the economic importance of chickpea (*C*. *arietinum* L.) seeds, it is important to understand the mechanisms underlying seed development for which a cDNA library was constructed from 6 day old chickpea embryos. A total of 8,186 ESTs were obtained from which 4,048 high quality ESTs were assembled into 1,480 unigenes that majorly encoded genes involved in various metabolic and regulatory pathways. Of these, 95 ESTs were found to be involved in ubiquitination related protein degradation pathways and 12 ESTs coded specifically for putative F-box proteins. Differential transcript accumulation of these putative F-box genes was observed in chickpea tissues as evidenced by quantitative real-time PCR. Further, to explore the role of F-box proteins in chickpea seed development, two F-box genes were selected for molecular characterization. These were named as *CarF-box_PP2* and *CarF-box_LysM* depending on their C-terminal domains, PP2 and LysM, respectively. Their highly conserved structures led us to predict their target substrates. Subcellular localization experiment revealed that *CarF-box_PP2* was localized in the cytoplasm and *CarF-box_LysM* was localized in the nucleus. We demonstrated their physical interactions with SKP1 protein, which validated that they function as F-box proteins in the formation of SCF complexes. Sequence analysis of their promoter regions revealed certain seed specific cis-acting elements that may be regulating their preferential transcript accumulation in the seed. Overall, the study helped in expanding the EST database of chickpea, which was further used to identify two novel F-box genes having a potential role in seed development.

## Introduction

Apart from being an important source of food for humans and animals, seeds provide protection and nourishment to developing embryo and permit it to remain quiescent at dormant stage until conditions become favourable for seedling development [1]. Exploring the process of embryogenesis at the molecular and cellular levels can provide insights into different aspects of early seed development including the developmental and metabolic regulation [2]. Expressed sequence tag (EST) has proved its usefulness in functional genomics approaches for gene discovery, large-scale expression analysis in various tissues and developmental stages and genome annotation in many plant species such as in *Arabidopsis* [2,3], wheat [4], rice [5] and legumes, such as *Medicago truncatula* [6,7] and soybean [8].

Chickpea represents the third most important pulse crop in the world (FAOSTAT 2011; http://faostat.fao.org). It has a genome size of 740 Mb and chromosome number 2n = 16 [9]. Chickpea seed is high in carbohydrates and proteins and also contains minerals and vitamins essential for human nutrition [9]. Due to its agronomical usefulness, the study of seed development in chickpea is of particular importance. A significant number of ESTs have been generated from root, leaf and pod of chickpea [10–12]. More recently, chickpea transcriptome has been sequenced using next-generation sequencing technologies [13–16]. However, transcripts from developing chickpea embryo remain uncharacterized. Thus, it becomes necessary to develop a dataset of genes expressing in early seed that can be used as a platform to identify the cellular processes and regulatory networks that underlie early seed development.

The ubiquitin/ proteasome pathway is the major regulatory mechanism for selective protein degradation in a wide variety of cellular processes including plant development [17]. It involves three enzymatic steps: (1) activation of ubiquitin by E1 enzyme (ubiquitin activating enzyme) in an ATP dependent manner, (2) transfer of activated ubiquitin to E2 (ubiquitin conjugating enzyme) and (3) transfer of ubiquitin to the target protein by E3 complex (ubiquitin protein ligase). SCF complex (**S**kp1, **C**ullin1, **F**-box containing complex) is one of the best characterized E3 ligases and is composed of Skp1, Cullin1, Rbx1 and F-box protein [18]. F-box proteins comprise a large family of proteins found in yeast, nematode, fish, human, mammals and plants [19]. These proteins share a conserved 40–50 amino acid long F-box motif. These interact with Skp1 via an N-terminal F-box motif and with their substrate via a C-terminal protein-protein interaction domain [20]. Thus, F-box proteins play a crucial role in targeting appropriate substrates to the SCF complex. Plants have been reported to contain the largest known number of F-box proteins with nearly 800 F-box proteins identified in *Arabidopsis* and 971 in rice [19]. Several of them have been extensively studied in recent years and are reported to be regulating a diverse range of cellular functions such as in plant growth and development, floral development, lateral root formation, leaf senescence, circadian rhythms, self incompatibility, defence, stress response, control of cell cycle, hormone signaling and seed germination, etc. [21]. Although various F-box genes and their functions have been discovered in many living organisms in previous years, till now there is very little information about F-box genes in chickpea [22] or F-box genes involved specifically in seed development. However, one report by Jain et al. [23] has implicated the probable role of F-box genes in seed development in rice on the basis of expression analysis. Therefore, efforts have been made in the present study to identify F-box genes having a potential role in chickpea seed development.

The objective of this study was therefore to generate the first set of chickpea ESTs from 6 DAA (days after anthesis) embryos which would serve as a foundation to investigate the regulation of genes during early seed development. Analysis of this dataset revealed a distinct set of ESTs that encoded genes involved in protein turnover. We analyzed the transcript accumulation patterns of F-box genes for elucidating their roles in Ub/26S proteasome-mediated protein degradation pathway during seed development. Two putative F-box genes were selected for further molecular characterization on the basis of their preferential accumulation in developing chickpea seed as well as the nature of C-terminal domains present. These were named as *CarF-box_PP2* and *CarF-box_LysM* depending on their C-terminal domains, PP2 and LysM, respectively. F-box genes having these domains at their C-terminal ends have not been characterized till date. This study represents an important step towards understanding the early stages of chickpea seed development.

## Materials and Methods

### Ethics statement

No specific permission was required for growing the plants in the NIPGR (National Institute of Plant Genome Research, New Delhi, India) field which is meant for research purposes and is not privately owned or protected. The study did not involve any endangered or protected species.

### Plant Material

*C*. *arietinum* cv. ICCV2 (single-podded, *kabuli*) was used for the construction of the chickpea embryo cDNA library. It was grown in the field conditions and monitored for flowering. Completely opened flowers were tagged [24] and seeds were collected 6 days after anthesis (DAA). Chickpea embryos were separated manually from the seeds by using forceps and kept on ice in a sterile eppendorf tube. Excised embryos were frozen in liquid nitrogen, and stored at -80°C. For gene expression analyses at different stages of seed development, flowers were similarly tagged and seeds collected at 5 DAA, 10 DAA, 20 DAA, 30 DAA and 40 DAA. Fully opened flower tissue was also collected from the field. The leaf, stem and root tissues were collected from the 2 week old chickpea seedlings grown in greenhouse.

### cDNA library construction and EST sequencing

Total RNA was extracted from isolated frozen embryos by LiCl method [25]. Double stranded cDNA was synthesised using the SMART cDNA Library Construction Kit (Clontech, USA) according to the manufacturer's protocol, and introduced into the TOPO TA Cloning vector (Invitrogen). This was transformed into *E*. *coli* DH5α competent cells. Successful recombinants were obtained by screening white colonies grown on Luria–Bertani (LB) medium supplemented with 100 μg/ml ampicillin, 20% (w/v) IPTG and 2% (w/v) X-gal. A total of 8184 recombinant clones were picked and colony PCR performed to determine their insert size. Those having insert size >500 bp were selected for plasmid isolation using alkaline lysis method [26].

About 5000 plasmids were sequenced by 5’ end single-pass sequencing on automated DNA capillary sequencer ABI PRISM 3730X (Applied Biosystems) using BigDye Terminator (Applied Biosystems) technology according to the manufacturer's instructions using M13 forward primer. M13 reverse primer was used for those sequences which could not amplify with M13 forward primer. All EST sequences were deposited in the GenBank database under the Accession numbers JK475124-JK475133, EX567519 and JK707012- JK711021.

### EST sequence processing, assembly and annotation

The ESTs were scanned and trimmed for vector sequences using NCBI’s VecScreen tool. Low quality and short (<100 bp) sequences were also removed. High-quality ESTs were assembled into contigs and singletons using Contig Assembly Program CAP3 [27].

The resulting contigs and singletons were then imported into the functional annotation and analysis tool Blast2GO [28] and were compared against the NCBI non-redundant (Nr) protein database BLASTX (e value cut-off of 1e-3). Subsequently, mapping of the ESTs was performed. Based on the results obtained, Gene Ontology (GO) annotation was performed with default settings. GO terms were assigned to each unigene and classified into 3 functional annotation categories- Biological Process, Cellular Component and Molecular Function. Finally, the assignment of the ESTs into metabolic pathways was done by KAAS (http://www.genome.jp/tools/kaas/) and KOBAS (http://kobas.cbi.pku.edu.cn/home.do).

For the detection of novel transcripts, the chickpea transcripts datasets available at CTDB (Garg et al. 2011a) were used for the BLASTN analysis (e-value cutoff of 10^–5^). For comparative analysis, the seed specific transcripts of *Arabidopsis* were downloaded from TAIR (http://www.arabidopsis.org/), soybean seed transcripts from SoyBase [29] and *Medicago* early seed and late seed library EST sequences from DFCI MtGI database (http://compbio.dfci.harvard.edu/tgi/cgi-bin/tgi/gimain.pl?gudb=medicago). The downloaded sequences were BLAST searched (BLASTN; e-value cutoff of 10^–5^) against the chickpea embryo transcripts.

### Real-time PCR analysis

Total RNA extractions from different vegetative tissues (flower, leaf, root and stem) as well as seeds at 5 developing stages (5 DAA, 10 DAA, 20 DAA, 30 DAA and 40 DAA) were performed by LiCl method. The cDNA was synthesized from 3 μg of DNase I-treated RNA using M-MLV reverse transcriptase (Clontech, USA) according to the manufacturer’s instructions. Primer pairs used in quantitative real-time PCR have been listed in S1 Table and were designed with the Primer Express software (Applied Biosystems, USA). All the quantitative real-time PCR reactions included 2 μl of cDNA, 200 nM of each primer and 10 μl of SYBER GREEN PCR Master Mix (Applied Biosystems) in a final volume of 20 μl. The following thermal cycle conditions were used with the ABI 7500 Real Time System (Applied Biosystems, USA): (1) incubation at 50°C for 2 m, (2) initial denaturation step of 95°C for 10 m and, (3) 40 cycles of 15 s at 95°C and 1 m at 60°C. EF1α was used as control [30]. All quantitative real-time PCR experiments were performed twice using two biological replicates and each reaction was run in triplicate. The relative gene expression levels were obtained by relative quantification (RQ) according to the 2^-ΔΔCt^ method [31].

### Isolation and cloning of *CarF-box_PP2* and *CarF-box_LysM*

The EST, F-box protein_7672 (GenBank accession no. JK710732) in the cDNA library constructed from 6 DAA embryos of chickpea was used as a template to obtain full length sequence by 5’ and 3’ RACE using SMART RACE cDNA Amplification Kit (Clontech, USA). The gene-specific primers, GSP1_7672 and GSP2_7672 (listed in S1 Table) were used to amplify the gene from cDNA of 10 DAA chickpea seed. The PCR products were cloned into pGEM-T Easy vector (Promega) and sequenced. The full length cDNA sequence was deduced by aligning and assembling the sequences of the EST, 5’ RACE and 3’ RACE products. The open reading frame (ORF) sequence was deduced by ORF Finder (http://www.ncbi.nlm.nih.gov/gorf/gorf.html).

To obtain the full length sequence for F-box protein_3599 (GenBank accession no. JK709226), the EST sequence was BLAST searched against the whole genome sequence of chickpea [32]. The CDS was predicted by GenMark (http://exon.gatech.edu/eukhmm.cgi) and FGENESH (www.softberry.com). Primers were designed using Primer3 program (http://frodo.wi.mit.edu/) [33] to amplify the putative CDS from total cDNA of chickpea 10 DAA seed. The PCR product obtained was purified and cloned into pGEM-T Easy vector and confirmed by sequencing.

### Sequence analysis

The obtained full length sequences were analyzed using bioinformatic tools at the websites http://www.expasy.org/, http://www.ncbi.nlm.nih.gov/ and Predictprotein (http://www.predictprotein.org/). Conserved domain analysis was performed using Pfam (http://pfam.sanger.ac.uk/) and SMART (http://smart.embl-heidelberg.de/). Homologous sequences were searched using BLASTX with default parameters at NCBI (http://blast.ncbi.nlm.nih.gov/Blast.cgi) for phylogenetic analysis. Multiple sequence alignment was performed using Clustal Omega (http://www.ebi.ac.uk/Tools/msa/clustalo/). Phylogenetic relationship tree was constructed using MEGA (ver. 5) [34] by Neighbour-Joining (NJ) method utilizing the p-distance method and complete deletion options. The reliability of the obtained tree was tested using a bootstrapping method with 1000 replicates. The homologs for *CarF-box_PP2* are as follows: *M*. *truncatula* (XP_003590890.1), *Glycine max* (XP_006588465.1), *Phaseolus vulgaris* (XP_007143948.1), *V*. *vinifera* (CBI38318.3), *P*. *trichocarpa* (XP_002324348.1), *A*. *thaliana* (NP_973399.1), *Zea mays* (NP_001150987.1), *Oryza sativa* (EAY87984.1), *Hordeum vulgare* (BAJ93930.1) and for *CarF-box_LysM*, *M*. *truncatula* [XP_003591114.1], *G*. *max* [NP_001240110.1], *P*. *vulgaris* [XP_007144727.1], *V*. *vinifera* [XP_002284540.1], *A*. *thaliana* [NP_564673.1], *H*. *vulgare* [BAJ88125.1], *Zea mays* [DAA50836.1], *O*. *sativa* [NP_001050989.1], *Triticum aestivum* [ADP02186.1].

### Protein structure modeling

Homology modelling for *CarF-box_LysM* and *CarF-box_PP2* was performed using Modeller 9.10 [35]. Templates used for *CarF-box_LysM* were 2DJPA (Chain A, The Solution Structure of the LysM domain of Human Hypothetical protein Sb145), 4I6JB (Chain B, A Ubiquitin Ligase-substrate Complex) and 1FQVA (Chain A, Insights into SCF Ubiquitin Ligases from the structure of the Skp1-Skp2 Complex) which showed 38%, 44% and 28% identity, respectively with *CarF-box_LysM*. For *CarF-box_PP2* templates used were 3hq2A, 1p9eA and 2odoA showing 29%, 29% and 28% sequence identity with *CarF-box_PP2*, respectively. The best models were selected on the basis of the DOPE scores and were refined and energy minimization was done using KoBaMIN [36] and quality of the models assessed by PROCHECK [37], to obtain a final model. Structures were visualized using PyMOL (http://www.pymol.org/).

### Southern Hybridization

Genomic DNA was isolated from chickpea leaves by CTAB method [38]. DNA (10 μg) was digested with Bgl II, Hind III, Sal I, Rsa I and Sty I, respectively for *CarF-box_PP2* and Bgl II, Sal I and Nco I, respectively for *CarF-box_LysM*. Digested DNA was separated on 0.8% (w/v) agarose gel, and transferred onto Hybond N nylon membrane (Amersham Biosciences, UK) in 20x SSC. Full length cDNA of *CarF-box_PP2* and *CarF-box_LysM* were used as probe to hybridize the respective blots. The probes were labelled with α-^32^P-dCTP using NEBlot Kit (NEB, USA). Pre-hybridization was performed at 60°C for 2 h in pre-hybridization buffer (0.1 M sodium phosphate buffer- pH-7.2, 10% SDS and 0.5 M EDTA), followed by hybridization with the labelled probe kept at the same temperature overnight. After hybridization, the blots were washed with 2X SSC, 0.1% SDS for 10 m at 60°C followed by washing with 1X SSC, 0.1% SDS for 10 m at room temperature [39]. The membranes were exposed to PhosphorImager screen (Amersham Biosciences, UK) for 24 h and the images were acquired by scanning the screen with Typhoon 9210 scanner (Amersham Biosciences, UK).

### Northern Hybridization

Total RNA (20 μg) from different stages of chickpea developing seed and vegetative tissues was isolated by LiCl method, fractionated by electrophoresis on denaturing formaldehyde 1.5% (w/v) agarose gel and then transferred to Hybond N nylon membrane (Amersham, UK). The full length cDNA of *CarF-box_PP2* and *CarF-box_LysM* were used as probes. A 250 bp cDNA amplicon of EF1α (Acc. No. AJ004960) was used to reprobe the membrane. Hybridization, washing and exposure of membrane to PhosphorImager screen were performed according to the protocol described for southern hybridization.

### Semi-quantitative PCR analysis

First-strand cDNA was synthesized from total RNA of chickpea 10 DAA seed as described for real-time PCR analysis. Gene-specific primers, FBPP2, FBLysM and EF1α (internal control) used for amplifying the genes are listed in S1 Table. PCR amplifications were performed in Mastercycler (Eppendorf). The PCR reaction (50 μl) contained 2 μl first-strand cDNA template, 5 μl 10x Titanium Taq PCR buffer (Clontech), 0.5 μM of each primer, 0.25 mM of dNTPs and 0.2 μl of 50X Titanium Taq DNA polymerase (Clontech). The following PCR conditions were used: initial denaturation at 95°C for 3 m, 30 cycles of denaturation at 95°C for 30 s, annealing at 60°C for 30 s and extension at 72°C for 1 m 30 s. Final extension was done at 72°C for 7 m. The amplified products were visualized by electrophoresis in 1.2% ethidium bromide stained agarose gels.

### Subcellular localization

The full length ORFs of *CarF-box_PP2* and *CarF-box_LysM* without termination codons were each inserted between the Nco I and Spe I sites on the pCAMBIA1302 vector using primers FBLysM _PCAM and FBPP2_PCAM (S1 Table), so as to create in-frame fusion constructs between the F-box gene and the GFP gene. The fusion constructs *p35S*::*CarF-box_PP2-GFP* and *p35S*::*CarF-box_LysM-GFP* as well as control GFP vector were transformed into *Agrobacterium* strain GV3101 and infiltrated into *Nicotiana tabacum* leaf according to the protocol by Sparkes et al. [40]. After the incubation period of 72 h in growth chamber, GFP fluorescence was detected and images recorded using TCS-SP-2 (Leica, Germany) scanning confocal microscope. GFP was excited at 489 nm with the argon laser. The experiment was repeated and at least three replicates were used each time.

### Yeast two-hybrid Assay

Protein-protein interactions were analyzed using the MATCHMAKER Gold yeast two-hybrid system (Clontech, CA) according to the manufacturer’s instructions. The coding regions of *CarF-box_PP2* and *CarF-box_LysM* were amplified by PCR and cloned into the DNA binding domain vector (pGBKT7, Clontech) to make the baits. SKP1_ ORF primers were designed to amplify the coding sequence of *CarSKP1* from the EST (acc no. JK710347) which was cloned into the activation domain vector (pGADT7, Clontech) to make the prey. Y2H Gold yeast strain (Clontech) was co-transformed with plasmids *pGBKT7*:*CarF-box_PP2* and *pGADT7*:*CarSKP1*. Similarly, co-transformation was carried out with plasmids *pGBKT7*:*CarF-box_LysM* and *pGADT7*:*CarSKP1*. The yeast cultures were plated on synthetic drop out (SD) medium lacking leucine and tryptophan to test for co-transformation. Colonies obtained were streaked on SD medium without leucine, tryptophan, adenine and histidine and supplemented with X-α-gal and aureobasidin to test for protein-protein interactions. Furthermore, colony PCR was performed to amplify the inserts in the vector using primers PGBKT7 and PGADT7. All the primers used have been listed in S1 Table.

### Promoter Analysis

A 2500 bp region upstream of start codon of *CarF-box_PP2* and 700 bp region upstream of start codon of *CarF-box_LysM* were obtained from the chickpea genome [32] by BLAST search. The sequences were subjected to *in-silico* analysis using PLACE (http://www.dna.affrc.go.jp/PLACE/signalscan.html) and PlantCARE databases (http://bioinformatics.psb.ugent.be/webtools/plantcare/html/), online tools for promoter prediction.

## Results

### Generation and sequencing of chickpea embryo ESTs

To get an insight into the genes involved in chickpea early seed development, embryos were isolated from 6 DAA chickpea seeds and a cDNA library was constructed. A total of 8186 recombinant clones were obtained which were screened by colony PCR to check the size of inserts. Around 5000 recombinant clones were found to have inserts above 500 bp in length and were selected for Sanger sequencing. The sequences obtained were trimmed to remove the vector sequences from the ESTs using the VecScreen tool of NCBI. Also, those sequences which had length <100 bp, were removed. This resulted in 4048 good quality sequences which were then assembled using CAP3 program into 1480 unigenes comprising of 443 contigs and 1037 singletons (S2 Table). The unigene length ranged from 100 to 1400 bp with an average size of 575 bp. The number of ESTs in the contigs ranged from 2 to 288. About 91.6% of the contigs had less than 10 ESTs with 210 having only 2 sequences.

### EST annotation and functional classification

**Fig 1:**
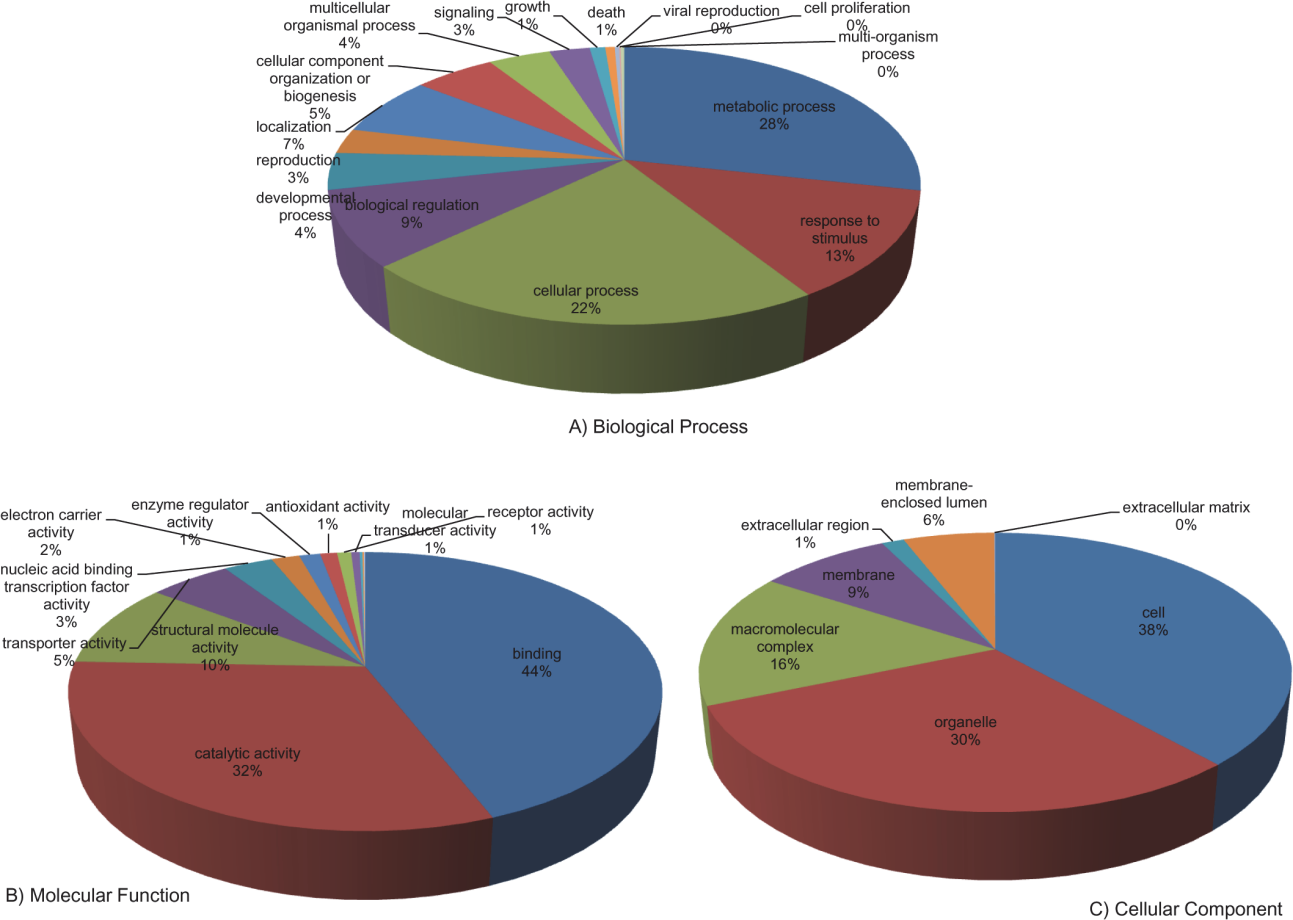
Functional classification. Distribution of gene ontology terms for the three categories: A) Biological Process, B) Molecular Function, and C) Cellular Component.

In order to assign putative functions to unigenes and analyse their involvement in different biological processes, Gene Ontology (GO) annotations were done using BLAST2GO program [28] which subjected the 1480 unigenes to BLASTX against NCBI Nr (non-redundant) protein database. A total of 1025 (69.2%) unigenes found significant hits with an e-value cut-off ≤ 10^–3^. Of the 1025 unique sequences with significant hits, 894 (60.4%) showed similarity to proteins of known function, 131 (8.85%) showed similarity to predicted proteins of unknown function (42 unigenes corresponded to ‘hypothetical protein’, 15 to ‘uncharacterized protein’, 57 to ‘protein’ and 17 to ‘unknown protein’), whereas 455 (30.7%) showed no significant similarity to any sequence contained in the Nr database. The GO annotation of the unigenes revealed mapping of 95.2% (976 out of 1025) of the unigenes showing a significant hit out of which 815 (55%) were successfully annotated with 4334 GO terms distributed among the three GO categories- Biological Process, Cellular Component and Molecular Function (Fig. 1). The unigenes were thus functionally classified with one or more ontologies with 672 (45.4%) of the 1480 unigenes been assigned GO terms associated with molecular functions, 663 (44.8%) were involved in a biological process, and 661 (44.6%) were cellular components. Under the category ‘Biological Process’, subcategories “metabolic process”, “cellular process”, “response to stimulus” and “biological regulation” accounted for 28%, 22%, 14% and 9% of the annotations, respectively. In the category ‘Molecular Function’, 44% of the unigenes annotations were grouped into the subcategory “binding” and 32% in the subcategory “catalytic activity”. Within the ‘Cellular Component’ category, 38% belonged to “cell” followed by 33% to “organelle” and 16% to “macromolecular complex”.

Unigenes were also annotated using KAAS (KEGG Automatic Annotation Server) and KOBAS (*KEGG Orthology Based Annotation System)*. KAAS results showed enrichment for the category metabolic pathways (98 unigenes), followed by the categories ribosomes (71), biosynthesis of secondary metabolites (40), oxidative phosphorylation (24), and protein processing in endoplasmic reticulum (18). KOBAS database showed similar results and the pathways were further characterized into subsets which has been summarized in S3 Table. Under the metabolism category, energy metabolism (30.5%), secondary metabolites (22.7%), carbohydrate metabolism (11.6%) and amino acid metabolism (11.2%) were major contributors. In the category of genetic information and processing, translation (72.8%) and folding, sorting, and degradation (18.9%) were the major sub-categories. In the category of environmental information processing, the majority were related to signal transduction (39.5%). Cell death (62.5%) constituted the majority of cellular processes category as opposed to cell growth (28.1%).

Additionally, the NGS based datasets of *desi*, *kabuli* and wild chickpea transcriptome available in Chickpea Transcriptome Database ([13]; http://www.nipgr.res.in/ctdb.html) were used for the BLASTN analysis. Of the 1480 unigenes, 1414 showed high homology to known chickpea transcripts. Thus, 66 putative novel chickpea transcripts were discovered. The 1480 unigenes were also BLAST searched against the datasets containing transcripts derived from seed tissues of *A*. *thaliana* (TAIR), *G*. *max* (SoyBase; [29]) and *M*. *truncatula* (The DFCI *Medicago truncatula* Gene Index) to find out the genes common between the different plant species during seed development. A total of 245 unigenes found match with *Arabidopsis*, 101 with soybean and 312 unigenes found homology with ESTs from *Medicago* seed tissues. A total of 20 transcripts were found to be common across the three species studied which comprised of ESTs encoding genes such as lipid transfer protein, cysteine proteinase, elongation factor, heat shock protein, NAC domain protein, defensin, among others.

### Most abundant ESTs

To identify genes that were highly expressed in the 6 DAA chickpea embryos, contigs that were built of the most abundantly occurring ESTs were identified. Transcripts frequently found in the library were genes associated with (i) cell growth and maintenance (histone H3.2, Ca^+2^ ATPase, chk1 checkpoint-like protein, proline-rich protein), (ii) stress and defence responses (defensin precursor, putative senescence-associated protein) (iii) transporters (early nodulin 93, nuclear transport factor), (iv) translation and post-translational modifications (DNA-directed RNA polymerase, ribosomal protein, translation elongation factor, polyubiquitin), (v) transcription factors (zinc finger family protein, AP2/ERF domain-containing transcription factor), and (vi) EST sequences without functional annotation. ESTs encoding histones were found to be most abundant. Highly abundant unigenes with >10 ESTs have been presented in S4 Table. ESTs encoding storage proteins were, as expected, almost nonexistent at this stage of seed development.

### ESTs involved in ubiquitination related protein degradation pathway

**Fig 2:**
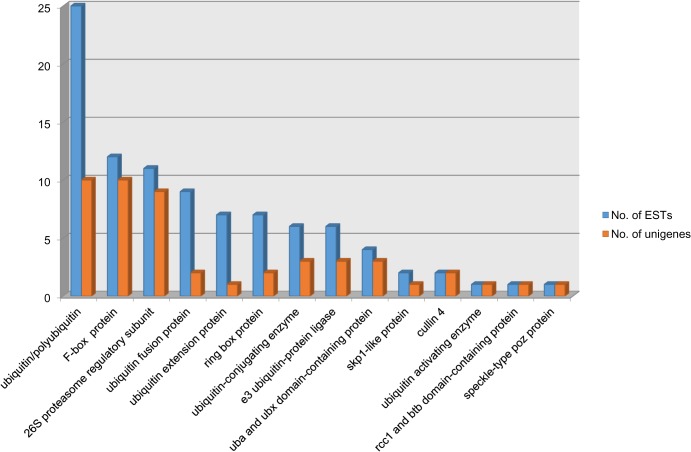
The categories of ubiquitin mediated protein degradation pathway transcripts. Distribution of ESTs and unigenes in each category is presented.

Interestingly, 49 unigenes (comprising of 95 ESTs) exhibited homologies with the genes involved in ubiquitination related protein degradation pathway (Fig. 2) suggesting that Ub-mediated protein degradation processes may play an important role in seed development. Occurrence of a significant number of transcripts encoding ubiquitin/polyubiquitin (25 ESTs), F-box proteins (12), 26S proteasome regulatory subunit (11), ubiquitin fusion protein (9), RING box protein (7), Skp1-like protein (2) and Cullin1 protein (2) among others suggests that SCF complex mediated 26S proteasome degradation pathway may be prevalent during seed development. For further analysis, we focused on ESTs encoding the F-box protein, a subunit of SCF complex involved in 26S proteasome degradation pathway and responsible for conferring specificity to the SCF complex for a substrate that has to be degraded. The 12 ESTs encoding putative F-box genes were assembled into 9 unigenes which have been listed in Table 1.

**Table 1:** List of ESTs encoding putative F-box genes.

**S. No.**	**Putative F-box gene**	**C-terminal domain**	**Accession No.**	**No. of ESTs**
1	Cyclin like F-box_484	-	EX567519	1
2	Tubby protein_661	Tub	JK707406	1
3	Kelch repeat containing F-box protein_1520	Kelch repeat	JK707984	1
4	F-box protein_3599	LysM	JK709226	1
5	Cyclin like F-box protein_4086	-	JK709422	1
6	F-box protein_7672	PP2	JK710732	1
7	PHD/F-box protein_430	PHD	JK710881	3
8	Tubby protein_7979	Tub	JK710918	1
9	skp1 interacting protein 15	-	JK710696	2

### Transcript profiles of putative F-box protein encoding genes

**Fig 3:**
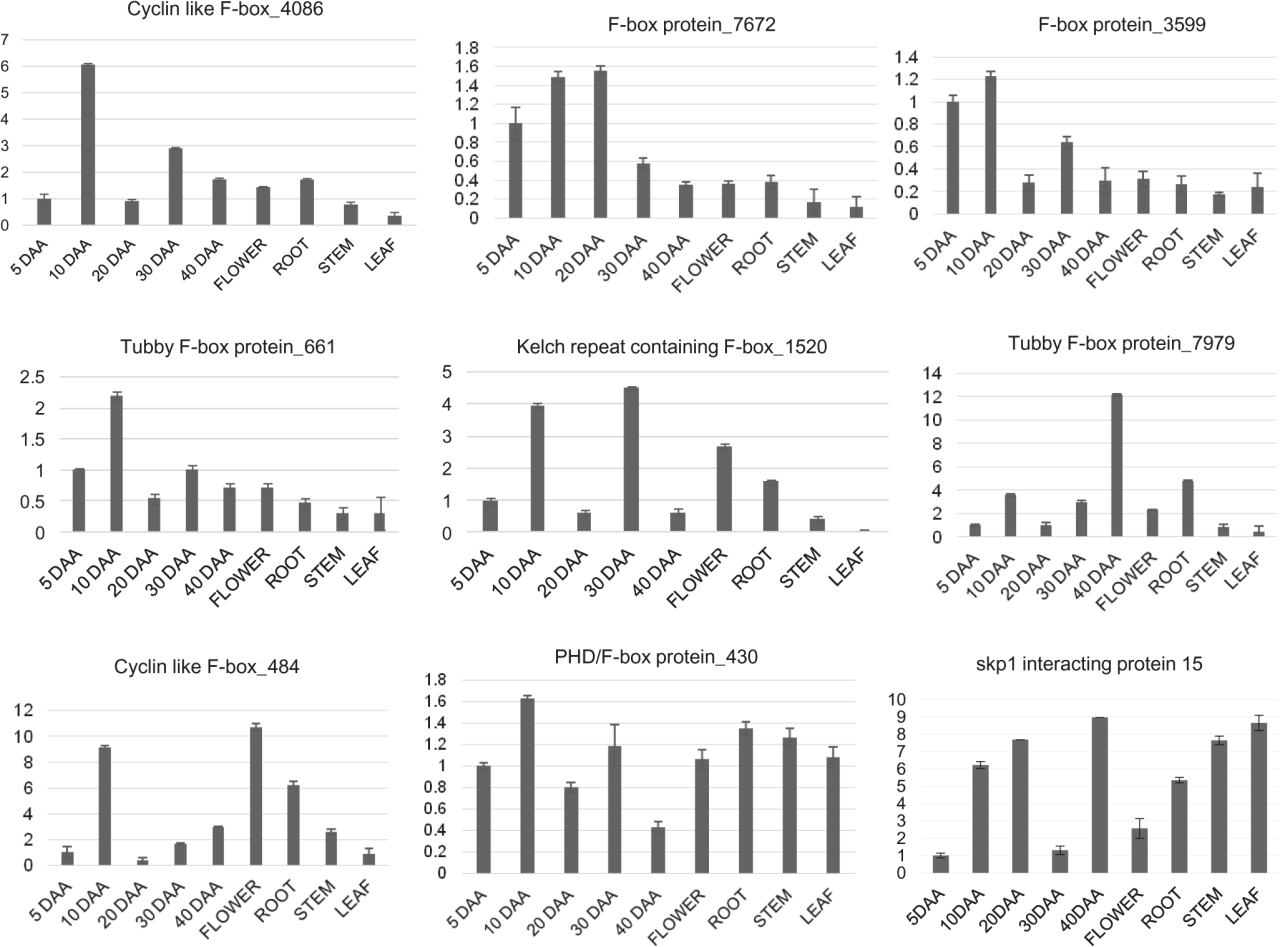
Transcript profiles of putative F-box protein encoding genes. The transcript accumulation patterns were observed in various chickpea organs including developing seeds at 5, 10, 20, 30 and 40 days after anthesis (DAA), flower, root, stem and leaf. The expression levels were obtained by normalization with chickpea EF1α. The error bars indicate standard deviations.

To investigate the physiological role, the transcript levels of the 9 F-box encoding genes obtained from the cDNA library were monitored across the different stages of chickpea seed development as well as in other vegetative tissues viz., flower, root, stem and leaf using quantitative real-time PCR (Fig. 3). Transcripts encoding Cyclin like F-box protein_4086 (Acc no. JK709422), F-box protein_3599 (Acc no. JK709226), F-box protein_7672 (Acc No. JK710732) and Tubby protein_661 (Acc no. JK707406) showed preferential expression in early stages (5–20 DAA) of seed development whereas, Kelch repeat containing F-box protein_1520 (Acc No. JK707984) and Tubby protein_7979 (Acc no. JK710918) showed higher expression in the later stages (30 and 40 DAA, respectively) of seed development. Cyclin like F-box_484 (Acc no. EX567519) expressed highly in flower whereas PHD/F-box protein_430 (Acc no. JK710881) showed almost equal level of expression in all the tissues. Transcripts encoding skp1 interacting protein 15 (Acc no. JK710696) showed ubiquitous expression in different chickpea tissues.

### Sequence analyses of *CarF-box_PP2* and *CarF-box_LysM*

The two ESTs encoding putative F-box proteins namely, F-box protein_7672 and F-box protein_3599 were chosen for detailed molecular characterization depending on their preferential transcript accumulation in the early stages of seed development as well as the C-terminal domains present. The putative F-box protein_7672 was designated as *CarF-box_PP2* (for *C* *icer* *ar* *ietinum* F-box protein containing PP2 domain) and F-box protein_3599 as *CarF-box_LysM* (for *C* *icer* *ar* *ietinum* F-box protein containing LysM domain).

**Fig 4:**
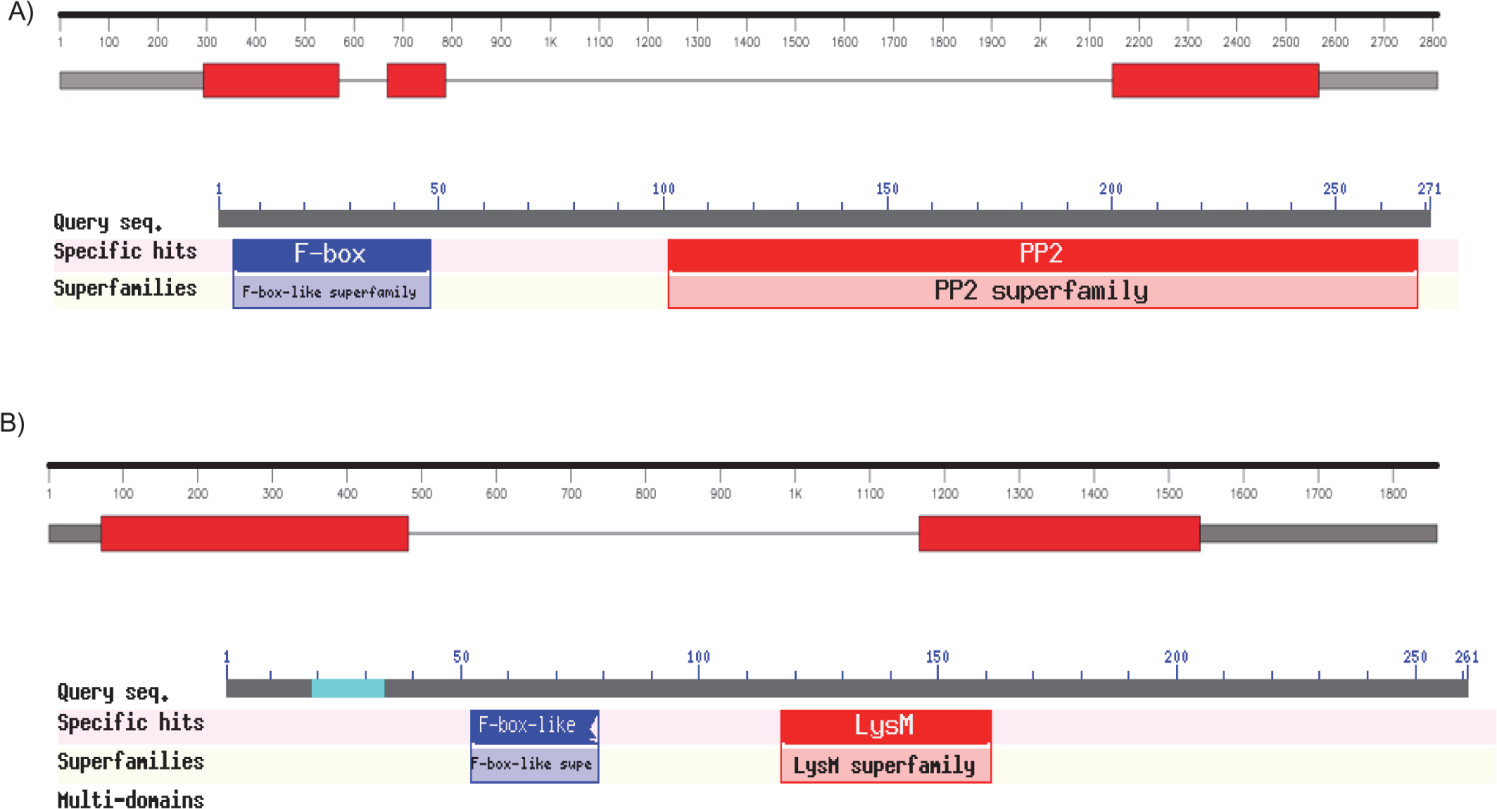
Gene structure of A) *CarF-box_PP2* B) *CarF-box_LysM*. Red boxes indicate exons, grey boxes are UTRs and lines between boxes indicate introns. Conserved domain organization as determined using NCBI conserved domain database (http://www.ncbi.nlm.nih.gov/Structure/cdd/wrpsb.cgi/).

RACE technology was used to obtain the full length sequence of *CarF-box_PP2* which was 1214 bp in length consisting of 147 bp 5’-untranslated region (5’ UTR), 816 bp coding region and 251 bp 3’-untranslated region (3’ UTR) (Fig. 4A). The full length of *CarF-box_PP2* has been deposited in NCBI GenBank with accession number JX294994. On comparing the cDNA sequence with the genomic sequence of the gene from the available chickpea genome [32], it was found to contain 3 exons and 2 introns and was present on chromosome 4. It encodes a protein of 271 amino acid length having a predicted molecular mass of 31.15 kDa with a calculated pI of 8.21 as calculated by Expasy tool. Analysis of the protein sequence using Pfam and SMART softwares revealed a conserved cyclin like F-box motif at the N-terminal (amino acid positions 4–47) which suggests that the gene encodes a putative F-box protein. Also, Phloem Protein 2 (PP2) domain was predicted at the C-terminal (amino acid positions 102–268) (Fig. 4A). BLASTX result showed homology of *CarF-box_PP2* with F-box proteins from other plant species viz., *M*. *truncatula* (71%), *L*. *japonicus* (69%), *G*. *max* (67%), *P*. *trichocarpa* (50%), *R*. *communis* (49%), *V*. *vinifera* (48%), *A*. *thaliana* (45%) and *O*. *sativa* (41%). The F-box domain of the gene was found to be highly conserved when aligned with the F-box motifs of its homologs using Clustal Omega (S1A1 Fig.). In order to find the phylogenetic relationship between *CarF-box_PP2* and F-box genes of other plants, a phylogenetic tree was constructed using the amino acid sequences of *CarF-box_PP2* and homologous sequences from various plants (S1A2 Fig.). The results showed that *CarF-box_PP2* was most closely related to the F-box gene of *M*. *truncatula*. Southern blotting analysis revealed that *CarF-box_PP2* is a single copy gene in the chickpea genome (S2A Fig.). Three bands were observed with chickpea genomic DNA digested with restriction enzymes, Rsa I and Sty I, which cut the gene at two sites, whereas only one band was observed when the gene was cut with restriction enzymes, Bgl II, Hind III and Sal I, the sites for which were not present within the gene.

A 1250 bp full length sequence of *CarF-box_LysM* was obtained by BLAST searching the 692 bp EST F-box protein_3599 against whole genome sequence of chickpea [32]. It contained a 5’UTR of 109 bp, 786 bp of coding region and 3’UTR of 355 bp (Fig. 4B). The full length of *CarF-box_PP2* has been deposited in NCBI GenBank with accession number JX294993. On comparing the cDNA sequence with the genomic sequence of the gene from the chickpea genome, it was found to contain 1 intron and was present on chromosome 4. The *CarF-box_LysM* gene encodes a protein of 261 amino acids with a calculated molecular mass of 29.16 kDa and an isoelectric point of 6.21. Analysis of the protein sequence using Pfam and SMART softwares revealed a conserved cyclin like F-box motif at the N-terminal (amino acid positions 44–84) which suggests that the gene encodes a putative F-box protein. Also, LysM domain was predicted at the C-terminal (amino acid positions 115–159) (Fig. 4B). BLASTX results showed that the gene exhibits 89% identity to *M*. *truncatula* F-box protein, 82% to *G*. *max*, 67% to *V*. *vinifera*, 65% to *P*. *trichocarpa*, 63% to *A*. *thaliana*, 62% with *R*. *communis* and 55% to *O*. *sativa*. The F-box domain was found to be highly conserved when aligned with its homologs (S1B1 Fig.). To understand the evolution of *CarF-box_LysM*, a phylogenetic tree was constructed based on full length amino acid sequences of *CarF-box_LysM* and 9 homologs of this protein from other plants (S1B2 Fig.). The results indicated that as expected the protein was most closely related to its homolog from *M*. *truncatula*. Southern blot analysis revealed that *CarF-box_LysM* is a single copy gene in chickpea genome (S2B Fig.).

### *CarF-box_PP2* and *CarF-box_LysM* proteins have conserved structures

**Fig 5:**
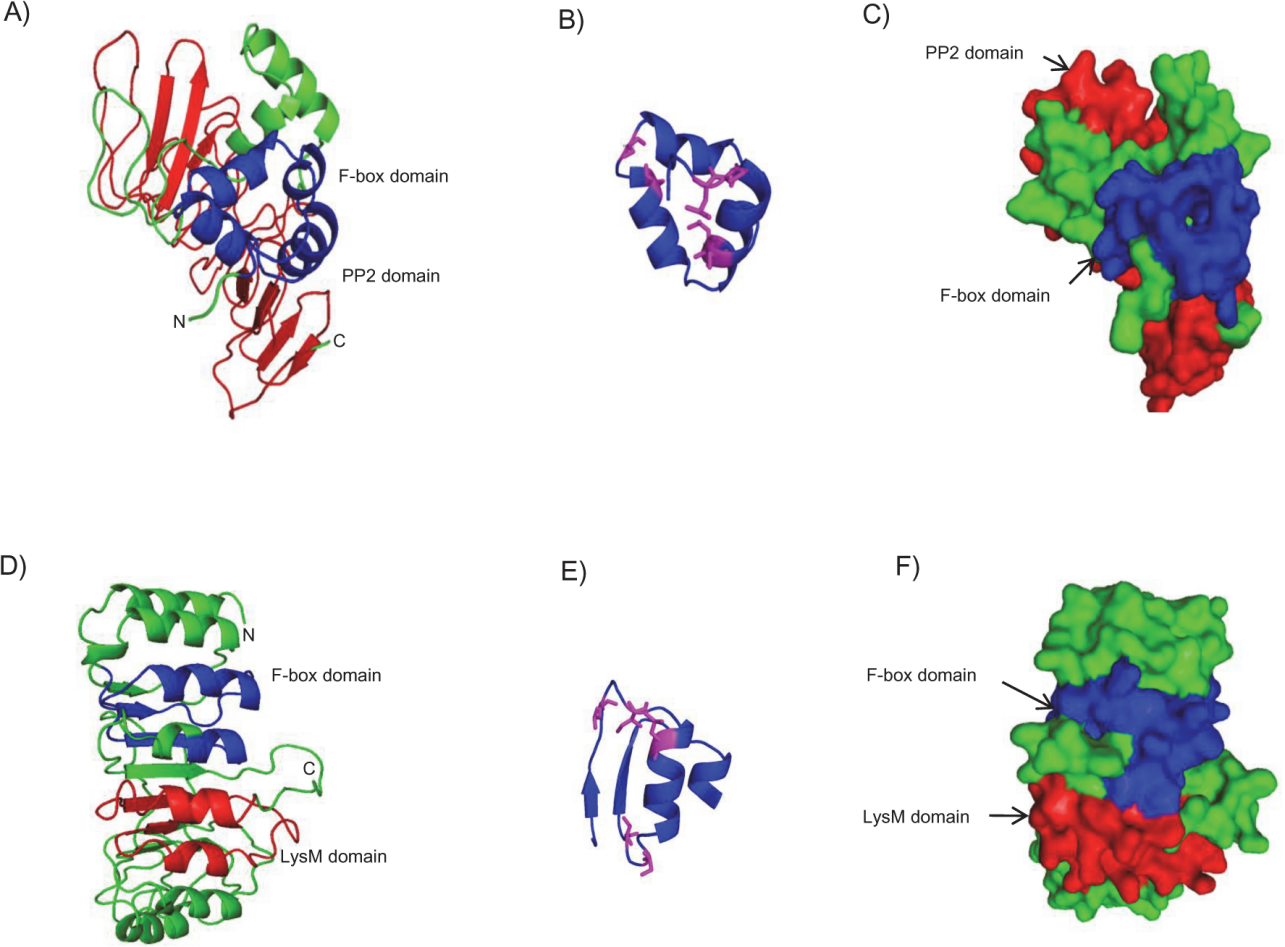
Structural models of *CarF-box_PP2* and *CarF-box_LysM*. The 3D structures are shown as (A,D) ribbon diagram and (C,F) surface view. The F-box domains are labelled and shown in blue colour. The C-terminal domains, PP2 and LysM are shown in red. Conserved residues in the F-box domains (B,E) are shown in pink.

To build structural models of *CarF-box_PP2* and *CarF-box_LysM* proteins, homology modelling using Modeller 9.10 was performed and the reliability as well as model quality of the final structural models was assessed with PROCHECK [37]. The Ramachandran plot generated by PROCHECK showed that the u/c angles of the majority of the residues were in the favoured regions, indicating that the predicted models had a good stereochemical quality. *CarF-box_PP2* showed 3-alpha helical F-box domain followed by a PP2 domain comprising of 8 parallel β strands (Fig. 5A–5C). PP2 domain was depicted by loops also, probably because it could not find structural homology with the templates in these regions. Two most highly conserved residues in the F-box domain were Leu7 and Pro8 along with others such as Ile15, Leu16, Ser27 and Ser30. On the other hand, the structure model of *CarF-box_LysM* consisted of two domains, N-terminal F-box-like domain and the C-terminal LysM domain. The F-box like domain consists of α-helices and parallel β-strands and LysM domain showing typical βααβ secondary structure where the two helices were present on the same side of an anti-parallel β sheet (Fig. 5D–5F). The most highly conserved residues in the F-box-like domain were Leu52, Ile55, Leu59, Ser68 and Val70. Overall, the structures of domains in *CarF-box_LysM* and *CarF-box_PP2* were found to be highly conserved.

### *CarF-box_PP2* and *CarF-box_LysM* show differential expression in seed tissues

**Fig 6:**
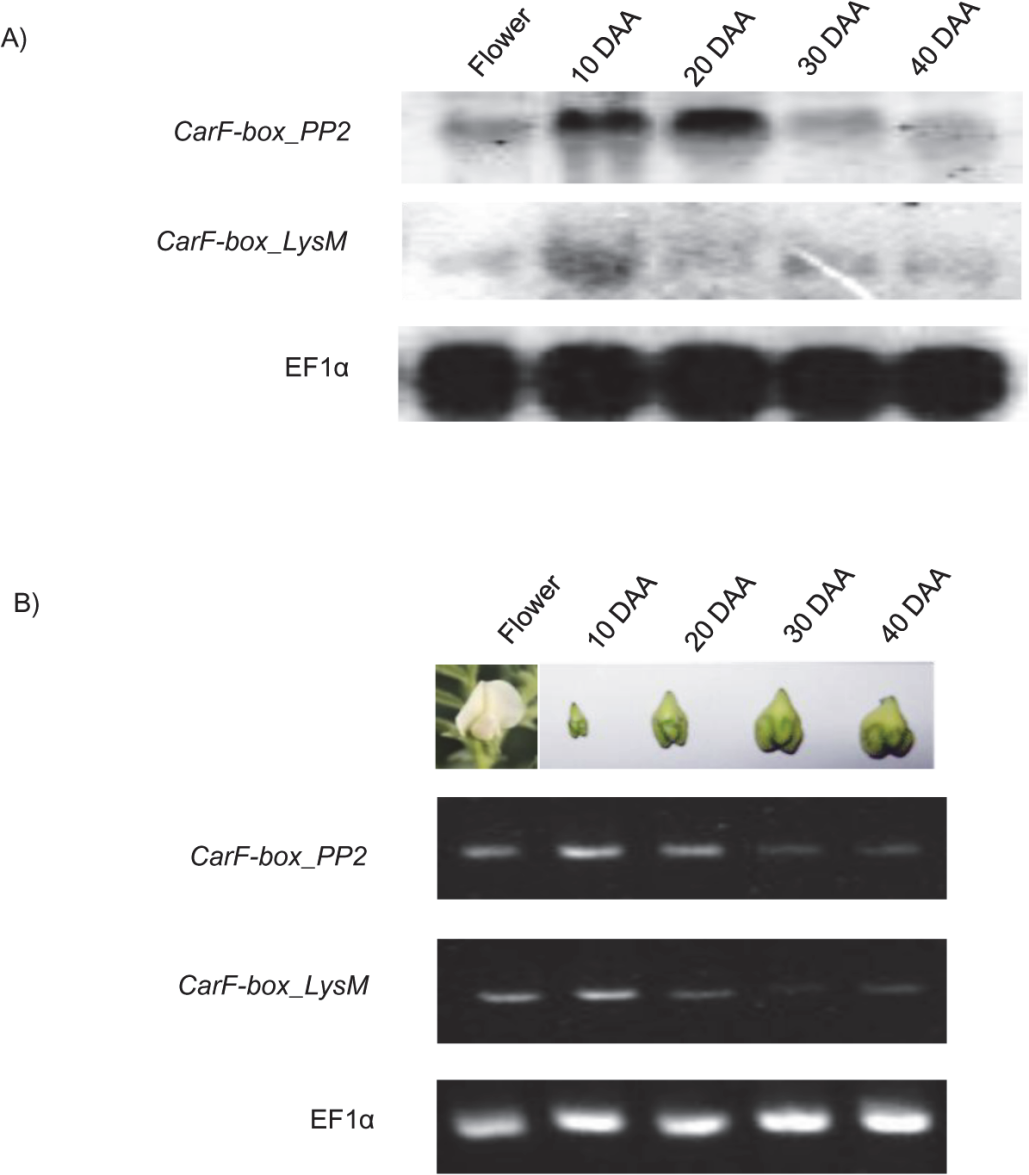
Expression analysis of *CarF-box_PP2* and *CarF-box_LysM*. A) Northern-blot analysis of the total RNA isolated from fully-opened flower and developing seeds (10 DAA, 20 DAA, 30 DAA and 40 DAA) of chickpea. Full-length *CarF-box_PP2* and *CarF-box_LysM* cDNA were used as probes. EF1α was used to reprobe the blot. B) Semi-quantitative PCR analysis. The cDNA was amplified using gene-specific primers for *CarF-box_PP2*, *CarF-box_LysM* and EF1α.

Northern blot analyses confirmed differential expression of *CarF-box_PP2* in the developing seed with more expression during the early stages of seed development (10 DAA and 20 DAA) (Fig. 6). Similarly, expression level of *CarF-box_LysM* was seen to be higher in early stage of seed development (10 DAA) and lower in later stages (20–40 DAA) (Fig. 6).

Semi-quantitative PCR was also performed to complement the results obtained by quantitative real-time PCR and Northern analyses. The results were fairly consistent showing similar trends in all the cases. Taken together, the results suggest that both the genes may be playing a key role during early stages of chickpea seed development.

### *CarF-box_PP2* is a cytoplasmic protein and *CarF-box_LysM* is a nuclear protein

**Fig 7:**
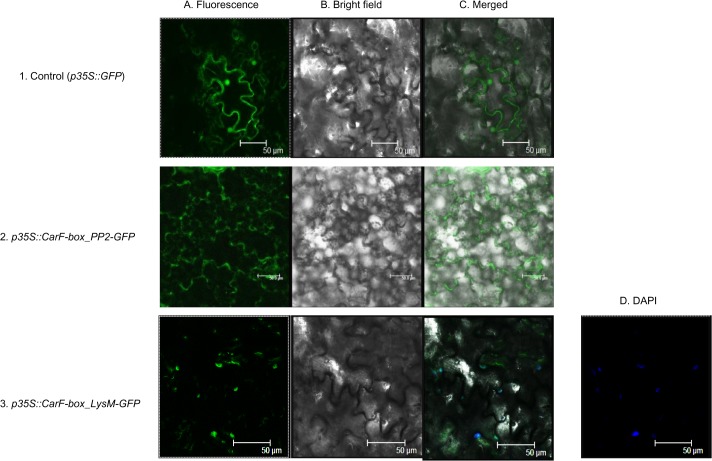
Subcellular localization of GFP-tagged *CarF-box_PP2* and *CarF-box_LysM* proteins. Transiently transformed tobacco leaf epidermal cells show enrichment of the *CarF-box_PP2* protein in the cytoplasm and *CarF-box_LysM* in the nucleus. A) Flourescence, B) Bright field, C) Merged and D) DAPI stained nuclei. 1) GFP only (Control) 2) *p35S*::*CarF-box_PP2-GFP*, 3) *p35S*::*CarF-box_LysM-GFP*. Scale bars = 50 μm.

*Agrobacterium* mediated transient transformation revealed the subcellular localizations of *CarF-box_PP2* and *CarF-box_LysM*. The control GFP was uniformly distributed throughout the cell. GFP signal of *CarF-box_PP2* was detected predominantly in the cytoplasm, whereas, *CarF-box_LysM*-targeted GFP accumulated in the nucleus. This was further confirmed by DAPI staining. Blue colour stained nucleus suggests that *CarF-box_LysM* is a nuclear protein (Fig. 7). The results correlated with the localization predictions made by PredictProtein tool which predicted that *CarF-box_LysM* has a nuclear localization signal DSLKRS at position 217 in the amino acid sequence.

### *CarF-box_PP2* and *CarF-box_LysM* interact with *CarSKP1*

**Fig 8:**
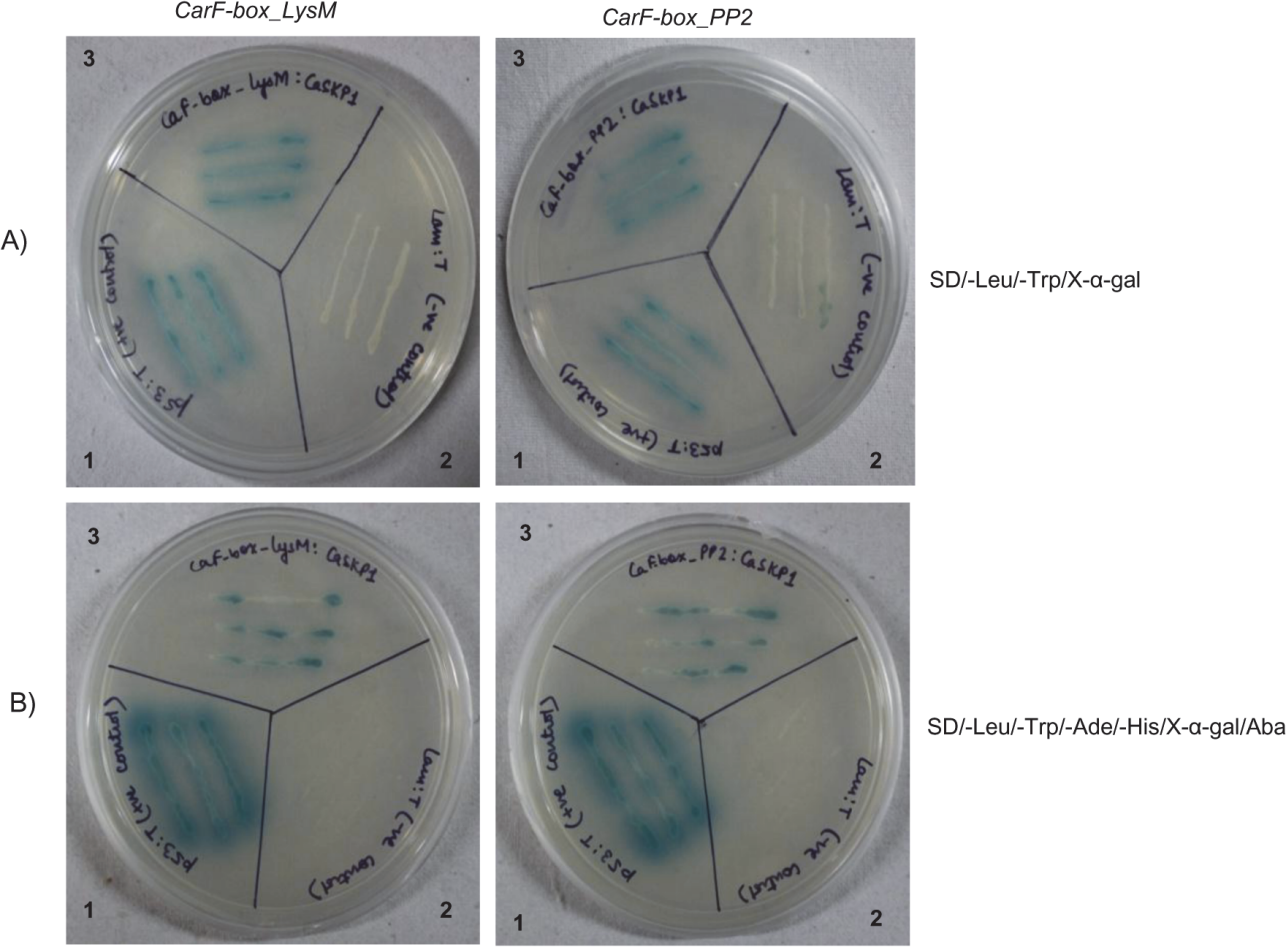
Interaction of *CarF-box* and *CarSKP1*. The interactions are shown in transformed Y2H Gold cells of yeast grown on SD/-Leu/-Trp/X- α-gal (A) and SD/-Ade/-His/-Leu/-Trp/X-α-gal/Aba (B). 1, pGBKT7-53+pGADT7-RecT (positive control); 2, pGBKT7-Lam+pGADT7-RecT (negative control); 3, pGBKT7-CarF-box+pGADT7-CarSKP1.

Yeast two-hybrid assays were performed using the Gal4 based Yeast two hybrid system to evaluate whether *CarF-box_PP2* and *CarF-box_LysM* function as components of SCF complex by interacting with *CarSKP1* (Acc. No. JK710347). Transformed yeast colonies (*pGBKT7*:*CarF-box_PP2* + *pGADT7*:*CarSKP1* and *pGBKT7*:*CarF-box_LysM* + *pGADT7*:*CarSKP1*) appeared blue on DDO/X/A and QDO/X/A after incubation at 30˚C for 4 days similar to the positive control (positive interaction between p53 and T-antigen) (Fig. 8). The interactions were further confirmed to be positive by rescuing the bait plasmids from yeast cells and growing bait on LB/Kan medium and prey on LB/Amp medium by transforming *E*. *coli* as well as by sequencing. Thus it was demonstrated that both the genes were able to interact with *CarSKP1* protein providing evidence that both *CarF-box_PP2* and *CarF-box_LysM* were indeed F-box proteins and could form SCF complexes.

### Promoters of *CarF-box_PP2* and *CarF-box_LysM* may be seed-specific

Because of the preferential expressions of *CarF-box_PP2* and *CarF-box_LysM* in early stages of developing seeds, attention was also focused on the promoter regions of both the genes. The promoter sequences were analyzed with the PlantCARE [41] and PLACE [42] softwares to predict the core promoter and *cis*-elements. A putative TATA box along with several CAAT boxes and 5’ UTR Py-rich stretches were identified in both the promoters. Several kinds of multi-copies of seed-specific promoter motifs, such as RY repeat motif, ACGT motif, SEF4 motif, -300 element, Amylase box, Pyrimidine box, CAREs, AACA motif and Skn-1 motif (Table 2; S3 and S4 Figs.) were observed. These seed-specific motifs in the promoters of *CarF-box_PP2* and *CarF-box_LysM* may be directing their preferential expression in the seed. However, the bioinformatics analysis is to be complemented and validated with experiments.

**Table 2:** Putative seed-specific *cis*-elements in the *CarF-box_PP2* and *CarF-box_LysM* promoter sequences.

***cis*-Element**	**Consensus**	**Motif position** ^a^ **in *CarF-box_PP2* promoter**	**Motif position** ^a^ **in *CarF-box_LysM* promoter**	**References**
-300 Core/ Prolamin box	TGTAAAG	-1179	-	[63,64]
-300 element	TGHAAARK	-112, -150, 12331, -1489	-609	[63,64]
AACA motif	AACAAAC	-664	-	[65]
ACGT element	ACGT	-31, -1519, -1597, -1962	-143	[65]
Amylase box	TAACARA	-779, -1096, -1789	+30	[66]
CAREs	CAACTC	-2167	-	[67]
DPBF Core	ACACNNG	-726	-	[68]
Pyrimidine box	CCTTTT	-2033, -2064, -2094	-	[69]
RY repeat	CATGCA	-493, -2208	-49	[61,62]
SEF4 binding site	RTTTTTR	-71, -455, -486, -1080, -1137, -1999	-259	[70]
Skn-1 motif	GTCAT	-10, -2073	-65	[71]

a: Position of the *cis*-elements upstream/downstream of transcription start site

## Discussion

Although chickpea serves as one of the most important agricultural crops worldwide, its seed providing a major source of protein for humans and animals, few studies have been dedicated to seed development. A large collection of ESTs from different chickpea tissues are available in public databases. In chickpea, at the time of initiation of this study, there were < 10000 ESTs available in the dbEST (as of February 2009) which have currently increased to 38097. However, more recently in the last two years 34760 *desi* [13], 43389 *kabuli* [15] and 37265 wild [16] chickpea transcripts obtained by next generation sequencing technology have become available. Of these only a small percentage corresponds to young pod or seed. Moreover till date, there are no ESTs available in the EST database or in the CTDB chickpea database that have been isolated from the chickpea embryo. In this study, we have for the first time generated the ESTs from 6 DAA chickpea embryo with the aim of gaining insights into chickpea early seed development. The present study reports the generation and analysis of 4048 ESTs from 6 DAA chickpea embryos which were assembled into 1480 unigenes. Interestingly, the functional profile of the unigenes expressed in chickpea embryos as revealed by GO analysis was quite similar to the patterns of expression described for developing seeds of *Arabidopsis* [3], *M*. *truncatula* [6,7], soybean [8], rice [5] and *G*. *max* [29]. Overall, in 6 DAA embryos, a large proportion of genes related to cell cycle regulation, chromosome organization and DNA processing, protein synthesis, transport, signal transduction, metabolism, defence and stress response, protein fate, energy and transcriptional and translational regulator activity were observed, reflecting the probable high cell division activity required by the developing embryo [43]. Production of energy via pathways such as glycolysis, TCA cycle, starch and sucrose metabolism, oxidative phophorylation, and carbon fixation are very important for the developing embryo [1,6,8] as was also evident from our data which was significantly enriched for GO terms and metabolic pathways related to carbon metabolism, including glycolysis, gluconeogenesis, starch biosynthesis, and fatty acid biosynthesis reflecting high rate of metabolism occurring in the highly differentiating and developing tissues of embryo.

A substantial number of ESTs encoding protein synthesis components along with ribosomal proteins as well as protein degradation machinery components reflected a high demand for protein synthesis and turnover in developing seeds. This shows the need to produce and subsequently degrade huge amounts of proteins in the actively developing seeds illustrating the high energy requirements of the embryo tissue. Several ESTs encoding proteins related to ubiquitin mediated proteasomal degradation pathway were found in our embryo cDNA library including polyubiquitin, F-box protein, RING box protein, ubiquitin fusion protein, etc. suggesting that regulatory mechanisms at the post-translational level are important during seed development. Importance of ubiquitin/proteasome related genes during seed development has been reported earlier [7,44]. It is highly likely that the accumulation of these ESTs may have a significant role in chickpea seed development that involves a series of cell division and cell proliferation events.

F-box protein, a subunit of SCF complex, is one of the components of the 26S proteasomal degradation pathway machinery which targets diverse substrates for ubiquitination and thereby regulates a wide-range of cellular processes [21]. One strategy to elucidate the possible involvement of F-box genes in processes such as seed development is to monitor their transcript levels across the developmental stages of chickpea seed. Therefore, quantitative real-time PCR analysis of F-box genes was carried out to reveal their transcript accumulation patterns in different vegetative tissues as well as in different stages of seed development. Accumulation of F-box protein_3599 and F-box protein_7672 transcripts in the young seed tissue suggest that they may be participating in an important aspect of chickpea seed development such as protein degradation during cell division and cell structure construction to ensure rapid availability of proteins to the developing embryo. However, further experiments are needed to demonstrate their role in seed development.

Several F-box genes have been cloned and characterized from *Arabidopsis* and rice but only one F-box gene has been identified in chickpea, i.e. the kelch repeat containing F-box protein *CarF-box1* [22]. A diverse array of protein-protein interaction domains that participate in substrate recognition have been identified in the C-terminal regions of F-box proteins [45]. Moreover, F-box proteins not only use protein–protein interactions but also protein–carbohydrate interactions for substrate recognition in the Ub/proteasome pathway [46–48]. Domain organization analyses in the present study revealed that F-box protein_7672 and F-box protein_3599 contain PP2 (Phloem protein 2) and LysM domain, respectively at their C-terminals. The domains, PP2 and LysM, have been demonstrated to be probably recognizing glycoproteins and structural glycans, respectively [49,50]. Although PP2 and LysM domain containing proteins without F-box domains have been studied structurally and functionally [49–53], there is little information about such proteins containing F-box domain at their N-terminal ends [46–48]. This led us to speculate whether PP2 and LysM domains have retained their substrate specificity in proteins having F-box domains as well or they function differently in the presence of F-box domain. Therefore, in this study, we have characterized two novel F-box genes from chickpea, *CarF-box_PP2* and *CarF-box_LysM*, which had PP2 (Phloem protein 2) and LysM domain respectively, at their C-terminals and which showed preferential transcript accumulation in early stages of seed development.

It has been well established that F-box proteins interact with SKP1 proteins in the SCF complex [54]. Our yeast two hybrid assay demonstrated that both the chickpea F-box genes interacted with *CarSKP1* suggesting that the chickpea proteins were active F-box proteins and constituted a part of an SCF complex. Their substrate specificity can be speculated based on their domain architecture. Phloem lectins or Phloem protein 2 proteins (PP2 proteins) were earlier thought to be only associated with structural phloem proteins. Acquisition of additional domains, such as F-box domain, through the course of their evolution, reflected the divergence from their only phloem function and addition of a new function to target protein for degradation [51]. Beneteau et al. [49] demonstrated the lectin properties of the PP2 domain in the *Arabidopsis* protein AtPP2-A1 and suggested that the PP2 recognizes either endogenous glycoproteins or glycosylated components of insects such as aphids. The FBA family of F-box proteins in mammals, having lectin-like properties, was proposed to target misfolded glycoproteins for proteasomal degradation [47,48]. In another study in tobacco, it was established that F-box proteins with a Nictaba domain are capable of interacting with glycosylated proteins of the plant cell [52]. The structural basis of recognition and ubiquitination of glycoproteins has been studied in detail [55]. Moreover, all the lectin domains crystallized till date has a structure consisting of β strands. Owing to the high structural similarity between PP2 domain of the *CarF-box_PP2* protein and the other lectin domains known, it can be hypothesised that plant F-box proteins with PP2 domain have a lectin activity, recognising a glycoprotein for degradation in plant cells. Moreover, glycosylation of the proteins take place in the endoplasmic reticulum (ER). Misfolded proteins are translocated back to the cytoplasm to be disposed off by ER-associated Ub/proteasome degradation pathway [55]. It is highly likely that *CarF-box_PP2* may be targeting a glycoprotein in the cytoplasm as suggested by its localization in the cytoplasm.

The LysM motif recognizes structural glycans such as peptidoglycan, chitooligosaccharides (chitin) and other structurally-related oligosaccharides [50]. Peptidoglycan is the major structural component of the bacterial cell wall, while, chitin is a component of fungal cell wall. It is possible that *CarF-box_LysM* proteins are involved in defence responses implicated by bacteria or fungi, by recruiting their cell wall components to the SCF complex for degradation via their C-terminal LysM domain, thus, contributing to plant immunity. The structures of LysM domains in various proteins studied in *E*. *coli* [53] and *Arabidopsis* [50] have been shown to have conserved architecture of βααβ form. Similar domain architecture observed in *CarF-box_LysM* suggests a similar role of LysM domain in F-box proteins.

Promoters are important genetic engineering tools which can be used for directing the expression of foreign genes in the seed. Thus, it is quite promising to develop new seed-specific promoters which can drive the target gene to express specifically in seeds thereby opening up avenues to enrich and enhance seed nutrients through transgenic approaches. Although most of the seed-specific promoters identified thus far are from genes encoding seed-storage proteins [56,57], other genes highly expressing in seeds during development may also be exploited for the isolation of seed specific promoters [58]. Several such seed-specific promoters have been isolated and characterized such as from soybean [59] and *Arabidopsis* [60]. Chickpea seed being economically important, identification of such promoters will be helpful in chickpea crop improvement by utilizing biotechnological approaches to target seed-specific expression of important genes [58]. The present study showed that *CarF-box_PP2* and *CarF-box_LysM* transcripts preferentially accumulated in chickpea seed. The spatial and temporal expression patterns for both the genes increased the possibility of their promoters being seed-specific. Online tools such as PLACE and PlantCare revealed that, besides the core elements such as TATA box and CAAT box, promoters of both the genes had several *cis*-elements involved in directing seed-specific expression, such as the Skn-1 motif and the RY element, among others as mentioned in Table 2, which have been demonstrated to have seed-specific promoter activity [61–71]. Based on these observations, the promoters can be selected as probable candidates to direct gene expression in seeds.

## Conclusions

In this study, attempts have been made to expand the EST database of chickpea through the construction of cDNA library from 6 DAA chickpea embryos which here served two functions—firstly it provided a new EST dataset which provided an opportunity for the functional dissection of gene expression during seed development. Secondly, the dataset helped in identification of genes playing an important role during seed development leading to the characterization of two novel F-box genes in chickpea. Based on the results, it could be suggested that the ubiquitin-mediated proteolysis mechanism involving *CarF-box_PP2* and *CarF-box_LysM*, had a potential role in chickpea seed development, although further studies are needed to elucidate their precise role in seed development.

## Accession Numbers

The nucleotide sequences of ESTs reported in this paper has been submitted to GenBank with accession numbers JK475124-JK475133, EX567519 and JK707012-JK711021. The coding sequences of *CarF-box_LysM* and *CarF-box_PP2* genes have been submitted to NCBI with accession numbers JX294993 and JX294994, respectively.

## Supporting Information

S1 Table Primers used in the study. (PDF)

S2 Table EST sequence and assembly statistics. (PDF)

S3 Table The distribution of the KEGG pathways. (PDF)

S4 Table Highly abundant genes in the cDNA library. (PDF)

S1 Fig Analysis of the deduced amino acid sequence of A) *CarF-box_PP2*, B) *CarF-box_LysM*. 1) Amino acid alignment of F-box domains of A and B with their homologs. Hyphens indicate gaps introduced to maximize the sequence alignment. Residues are highlighted according to the degree of conservation. 2) Phylogenetic tree constructed using *CarF-box_PP2* and *CarF-box_LysM* sequences from chickpea and their homologs from other plant species. The phylogenetic tree was constructed with MEGA5; bootstrap values are in percentages
(PDF)

S2 Fig Genomic Southern blot analysis of A) *CarF-box_PP2*, B) *CarF-box_LysM*. Genomic DNA (10 μg) was digested with restriction enzymes depicted in respective lanes, separated on 0.8% (w/v) agarose gel and transferred onto Hybond-N nylon membrane. Full length cDNAs of A and B were labeled with α-^32^P-dCTP as probe.
(PDF)

S3 Fig Promoter region of *CarF-box_PP2*. The seed specific *cis*-acting regulatory elements are shown in colored blocks. Region underlined is the TATA box. TSS (transcription start site) is depicted by bold larger font.
(PDF)

S4 Fig Promoter region of *CarF-box_LysM*. The seed specific *cis*-acting regulatory elements are shown in colored blocks. Region underlined is the TATA box. TSS (transcription start site) is depicted by bold larger font.
(PDF)
