# Supplementary material for: A New Set of ESTs from Chickpea (Cicer arietinum L.) Embryo Reveals Two Novel F-Box Genes, CarF-box_PP2 and CarF-box_LysM, with Potential Roles in Seed Development
Source: PLoS One. 2015 Mar 24;10(3):e0121100. doi: 10.1371/journal.pone.0121100 (PMC4372429; doi:10.1371/journal.pone.0121100)
Supplement: S1 Table — (PDF) [file pone.0121100.s001.pdf]

S1 Table Primers used in the study

Primer name	Primer sequence
**Real-time PCR primers**	
Cyclin like F-box_484	F: GGTGAACTCGGTGTCAAAGAATC
	R: CAATACTAGACAAGGGCCCAACA
Cyclin like F-box_4086	F: CCAGTTTGGGTTGTAGGCTTTT
	R: CGGTACAGCAACACGAACTAGTG
F-Box protein_3599	F: CCTACTTGCCTACCCCACTGAA
	R: GTGATCCTCGAGCTGCTCTTG
F-Box protein_7672	F: TGCCTTGTCCCCAAATACTCA
	R: TCCCCAGTCACCGATCATGT
Kelch repeat containing F-box_1520	F: CCAGCCTCGGAGAGATTGC
	R: GAGTCGAGGATACGTCCCTATGA
PHD/ F-box protein_430	F: AGAAGATGAGCACAGCGAAACA
	R: CAGCCAATCCAAAACTCATCTG
Tubby F-box_661	F: TTTGGAAAAGTTGGAAAGGATGTAT
	R: CAAATGCTTCAAAGGCAGAGATT
Tubby F-box_7979	F: GTAACTTTGAGGCAGGGCTTGT
	R: TCTCCTTGGACCTCTTGATTTCA
Skp1 interacting protein 15	F: ACGCGGGTAATCAAGGACA
	R: TCCTTCGGAAGCACTTAAATCC
**RACE primers**	
GSP1_7672 (5' RACE)	GAAGGGAGAAAGCGATTCCAGACAGC
GSP2_7672 (3' RACE)	GGAAGATAAACGCCGTTGCCTTGTCC
**ORF primers**	
FBPP2	F: ATAATGACAGAGATTCATCAATTGCCG
	R: CCCCTAGTGAATGTTGCTTAATTATCTTCCTT
FBLysM	F: CCGAATGGGTTGTTGCTGCGATGA
	R: GGTTAGGAATGACCTACTTGCCTACCCCACT
SKP1	F: CATGTCTTCAACAAGGAAATTCACC
	R: CACTTTGCAAATCTCTGTGTGAGA
**Yeast two hybrid primers**	
SKP1_PGADT7	F: CGTCCCGGGCATGTCTTCAACAAGGAAAG
	R: CGTCTCGAGCATCTTTCAAATGCCCATTG
FBLysM_PGBKT7	F: CGTCCCGGGATGGGTTGTTGCTGCGAT
	R: CGTGTCGACAAGGTTCGGAATGACCTACTTGC
FBPP2_PGBKT7	F: CGTCCATGGTAATGACAGAGATTCATCAATTGCCG
	R: CGTGTCGACCTTGATTATCTTCCTTAAGCCTTACTTCGAT
PGADT7_insert	F: GATGATGAAGATACCCCACCAAAC
	R: CGGGGTTTTTCAGTATCTACGATT
PGBKT7_insert	F: CGGAAGAGAGTAGTAACAAAGGTCA
	R: CCCCAAGGGGTTATGCTAGTTATG
**Subcellular localization primers**	
FBLysM _PCAM	F:CGTAGATCTGAATGGGTTGTTGCTGCGAT
	R: CGTACTAGTGGTTCGGAATGACCTACTTGCCT

FBPP2_PCAM	F: CGTAGATCTTAATGACAGAGATTCATCAATTGCCG
	R: CGTACTAGTGTGAATGTTGCTTGATTATCTTCCTTAAG
PCAMBIA1302_insert	F: TTGATGTGATATCTCCACTGACGTA
	R: ACGGAACAGGTAGTTTTCCAGTAGT
**Expression Analysis primers**	
EF1α	F:TTAAGTCCGTTGAGATGCACCA
	R:CCACAACCATAGGCTTTGTTGG
