# Supplementary material for: A New Set of ESTs from Chickpea (Cicer arietinum L.) Embryo Reveals Two Novel F-Box Genes, CarF-box_PP2 and CarF-box_LysM, with Potential Roles in Seed Development
Source: PLoS One. 2015 Mar 24;10(3):e0121100. doi: 10.1371/journal.pone.0121100 (PMC4372429; doi:10.1371/journal.pone.0121100)
Supplement: S2 Table — (PDF) [file pone.0121100.s002.pdf]

**S2 Table** EST sequence and assembly statistics

| **Feature** | **Value** |
|---|---|
| Total no. of clones sequenced | 5000 |
| No. of high-quality sequences | 4048 |
| Avg. length of high-quality ESTs | 575 bp |
| No. of Contigs | 443 |
| No. of Singletons | 1037 |
| No. of Unigenes | 1480 |
