# Supplementary material for: A New Set of ESTs from Chickpea (Cicer arietinum L.) Embryo Reveals Two Novel F-Box Genes, CarF-box_PP2 and CarF-box_LysM, with Potential Roles in Seed Development
Source: PLoS One. 2015 Mar 24;10(3):e0121100. doi: 10.1371/journal.pone.0121100 (PMC4372429; doi:10.1371/journal.pone.0121100)
Supplement: S3 Table — (PDF) [file pone.0121100.s003.pdf]

S3 Table The distribution of the KEGG pathways

	**Pathway**	**Total no. of unigenes**	**Percent of unigenes (%)**	**Percent of categories (%)**
**Metabolism**	Carbohydrate metabolism	27	1.8	11.6
	Energy metabolism	71	4.7	30.5
	Lipid metabolism	26	1.7	11.2
	Nucleotide metabolism	13	0.8	5.6
	Amino acid metabolism	23	1.5	9.9
	Glycan biosynthesis and metabolism	2	0.1	0.8
	Metabolism of cofactors and vitamins	18	1.2	7.7
	Biosynthesis of secondary metabolites	53	3.5	22.7
**Genetic Information Processing**	Transcription	7	0.4	3.8
	Translation	131	8.8	72.8
	Folding, sorting, and degradation	34	2.3	18.9
	Replication and repair	8	0.5	4.5
**Environmental Information Processing**	Membrane transport	14	0.9	32.6
	Signal transduction	17	1.1	39.5
	Signaling molecules and interaction	12	0.8	27.9
**Cellular process**	Cell growth	9	0.6	28.1
	Cell cycle	3	0.2	9.4
	Cell death	20	1.3	62.5
**Organism systems**	Immune system	5	0.3	20
	Nervous system	3	0.2	12
	Development	6	0.4	24
	Environmental adaptation	11	0.7	44
