# Supplementary material for: A New Set of ESTs from Chickpea (Cicer arietinum L.) Embryo Reveals Two Novel F-Box Genes, CarF-box_PP2 and CarF-box_LysM, with Potential Roles in Seed Development
Source: PLoS One. 2015 Mar 24;10(3):e0121100. doi: 10.1371/journal.pone.0121100 (PMC4372429; doi:10.1371/journal.pone.0121100)
Supplement: S4 Table — (PDF) [file pone.0121100.s004.pdf]

S4 Table Highly abundant genes in the cDNA library

Contig	Homology	No. of ESTs	% Identity	E-Value	GENBANK Accession no.
Contig130	histone H3.2	288	100	7.66E-69	JK707657
Contig433	putative Ca^{2+}-ATPase	127	85	3.07E-42	JK710912
Contig 14	Hypothetical protein	98	60	1.20E-08	JK707201
Contig438	defensin	40	87	1.08E-18	JK710950
Contig374	senescence-associated protein	32	78	1.74E-86	JK709716
Contig213	hypothetical protein	31	100	1.76E-10	JK707141
Contig156	alcohol dehydrogenase 1	21	96	0	JK707613
Contig161	Unknown protein	19	66	5.01E-38	JK708464
Contig368	early nodulin 93	18	95	2.95E-33	JK708760
Contig 64	Nuclear transport factor 2	17	90	4.57E-62	JK708820
Contig 181	40s ribosomal protein s14	17	96.6	5.47E-78	JK708618
Contig 69	zinc finger protein	16	68	2.75E-78	JK707692
Contig 12	No significant similarity	16	-	-	JK707147
Contig 173	GTP-binding protein	15	95.8	2.39E-127	JK708551
Contig 344	hypothetical protein	15	91.5	1.11E-33	JK710124
Contig 395	chk1 checkpoint-like protein	15	84.25	6.69E-42	JK710630
Contig 2	No significant similarity	14	-	-	JK707053
Contig 40	translation elongation factor	14	99	0	JK707421
Contig 106	conserved hypothetical protein	14	69.8	1.03E-68	JK707569
Contig 387	proline-rich protein	14	47.5	1.30E-04	JK707027
Contig 186	Polyubiquitin	13	99	2.83E-122	JK708690
Contig 77	60s acidic ribosomal protein p1	13	85.4	3.27E-25	JK708331
Contig 362	TPR domain protein	13	75.5	9.34E-94	JK710341
Contig 18	No significant similarity	12	-	-	JK707226
Contig 364	Unknown protein	12	54	4.18E-07	JK710350
Contig 16	s-adenosylmethionine synthetase	11	97	0	JK707217
Contig 13	ribosomal protein s8e	11	86.5	8.30E-103	JK709694
Contig 27	60s acidic ribosomal protein	11	83.3	3.66E-26	JK707304
