# Supplementary material for: A New Set of ESTs from Chickpea (Cicer arietinum L.) Embryo Reveals Two Novel F-Box Genes, CarF-box_PP2 and CarF-box_LysM, with Potential Roles in Seed Development
Source: PLoS One. 2015 Mar 24;10(3):e0121100. doi: 10.1371/journal.pone.0121100 (PMC4372429; doi:10.1371/journal.pone.0121100)
Supplement: S1 Fig — 1) Amino acid alignment of F-box domains of A and B with their homologs. Hyphens indicate gaps introduced to maximize the sequence alignment. Residues are highlighted according to the degree of conservation. 2) Phylogenetic tree constructed using CarF-box_PP2 and CarF-box_LysM sequences from chickpea and their homologs from other plant species. The phylogenetic tree was constructed with MEGA5; bootstrap values are in percentages (PDF) [file pone.0121100.s005.pdf]

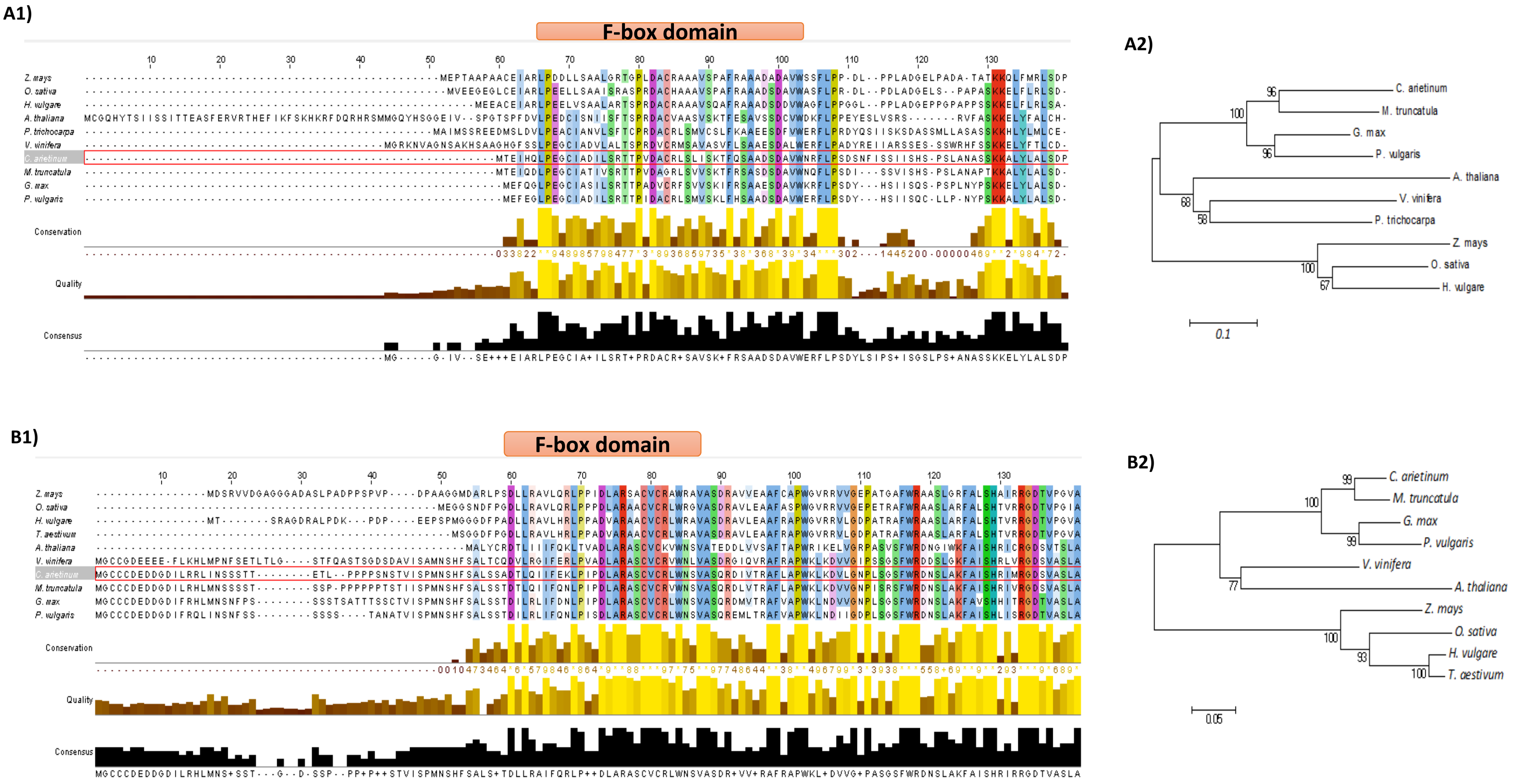

S1 Fig. Analysis of the deduced amino acid sequence of a) *CarF-box_PP2,* b) *CarF-box_LysM*

1) Amino acid alignment of F-box domains of A and B with their homologs. Hyphens indicate gaps introduced to maximize the sequence alignment. Residues are highlighted according to the degree of conservation

2) Phylogenetic tree constructed using *CarF-box_PP2* and *CarF-box_LysM* sequences from chickpea and their homologs from other plant species. The phylogenetic tree was constructed with the MEGA5; bootstrap values are in percentages
