# Supplementary material for: A New Set of ESTs from Chickpea (Cicer arietinum L.) Embryo Reveals Two Novel F-Box Genes, CarF-box_PP2 and CarF-box_LysM, with Potential Roles in Seed Development
Source: PLoS One. 2015 Mar 24;10(3):e0121100. doi: 10.1371/journal.pone.0121100 (PMC4372429; doi:10.1371/journal.pone.0121100)
Supplement: S2 Fig — Genomic DNA (10 μg) was digested with restriction enzymes depicted in respective lanes, separated on 0.8% (w/v) agarose gel and transferred onto Hybond-N nylon membrane. Full length cDNAs of A and B were labeled with α-32P-dCTP as probe. (PDF) [file pone.0121100.s006.pdf]

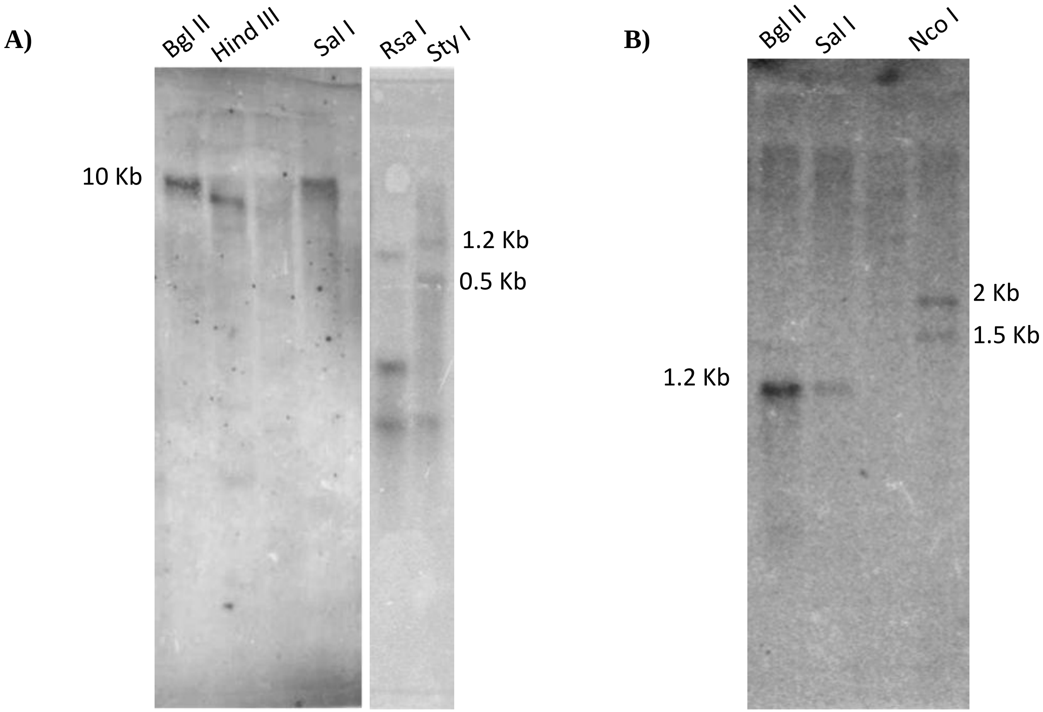

S2 Fig. Genomic Southern blot analysis of A) *CarF-box_PP2*, B) *CarF-box_LysM*. Genomic DNA (10 µg) was digested with restriction enzymes depicted in respective lanes, separated on 0.8% (w/v) agarose gel and transferred onto Hybond-N nylon membrane. Full length cDNAs of A and B were labeled with α-^{32}P-dCTP as probe.
