# Supplementary material for: A New Set of ESTs from Chickpea (Cicer arietinum L.) Embryo Reveals Two Novel F-Box Genes, CarF-box_PP2 and CarF-box_LysM, with Potential Roles in Seed Development
Source: PLoS One. 2015 Mar 24;10(3):e0121100. doi: 10.1371/journal.pone.0121100 (PMC4372429; doi:10.1371/journal.pone.0121100)
Supplement: S3 Fig — The seed specific cis-acting regulatory elements are shown in colored blocks. Region underlined is the TATA box. TSS (transcription start site) is depicted by bold larger font. (PDF) [file pone.0121100.s007.pdf]

-2353 CAGAAAATGAGAGAAAAGGGCCGAAGGGTCCCTCAAATATTAGCATGAAT

-2303 TAATTGAAGGACCCCCCACGGGAGGGGAGCAAAGGCAACAGGAGTGGCAA

-2253 GGAGATTAAAAGGGAAATATGAAAACAAGAGAAGAAGGAATTAGCCATGC

-2203 ATGGGTGCAATGAACAAAAATGATGATGTTTCCTCACAACTCTCTCCGAG

-2153 AGAAAAGGAGGATGTATGAATGGACTTAATTCATTATCATTGTAGGCTAC

-2103 TAATTCCACCCTTTTGCCTTCCAATTACATGTCATCCACCCTTTTTTCTT

-2053 AGCTTCATTGCTTAATTAAGCCTTTTTTTCTTTTTGTTTGCTTTTTAATT

-2003 ATGCGTTTTTACATTACTATTTAGTATATGGTTGGTTATTTACGTTGATT

-1953 TCTGATTTTCATCTTAAAACAACATGTGGAACAAATCCCTTTACTATGTC

-1903 TTTTCACAAGTTCAATCAATAACATTTGTTGGGAAAAATCAATGACTAAG

-1853 GGAGTATCAGTAAGTAATGTATAAATTCATACTCAAATTAATGTCTAAGC

-1803 CATCCAAGAAATTATAACAAAAAATTAGATAAATTAATGAAGATATAGTC

-1753 AGTTTAATATTTTACATGAAGATATATTAATATTCTTAATATATATATTC

-1703 ATATGCAAATAGGTAGTTAAAAAATAGTAAATATCTAAATTATTATTCTT

-1653 TTTTTTTACCAAAGAAACTCAAATTATTTCTCATAAGGGAAACTAAGCAA

-1603 AATTTTACGTGGTCAAATTAAAGTGAAAGTAAAATTGTGGAATAATAGTA

-1553 CACAATTAATAATGGAGTTTGGATAAATATGTCTACGTCTTCAAGCTTTT

-1503 GCATATCTTTATATTGAAAAAGAAAAATTTACGCAAAGTGTTTACAAACA

-1453 TTCACTATCAAAAATCAATTACACTCAAGAATGTCACTCATAGAAATCTA

-1403 TCTAGCTCTCTTGATATTCTCTCAATGTTTGAATTCTCTTTAAGTTGGAA

-1353 TGAATTTCACTCTTCATTCATCACCTCTATTTATAGGTGATTTAGATCAC

-1303 TTTTTTCTTACTTGGTTACTAAGTTTACTTAGGCTCCGTTTGATAAAATT

-1253 AAGCTATCAACTAGTTGATAGATGAAAAGGTGACTGATAGCTTAAAAGCT

-1203 AATTGATAACTGATGACTTATAGTTGTAAAGCTAATTAATTGAGATTAAA

-1153 ATGTTTGGTAAATTTAGTTTTTAACAATATAAAATGACATGATTATTTAA

-1103 TTTAAAATAACAAAAAATTTGACATTTTTATCCAATCTTCTAGCTATTGT

-1053 TTTAGTCGATCTATTTTTTTTTTTGTACTCAATCAATTTTCCTACTTCAT

-1003 TTTATTTCTTATCTTTTCTTCTTCTCCATGTTCTTAGACTTTATTTCAAA

-953 TATTTTCTTCCTCTTCATATCGCTAGTTAGTTGATGTTATCTTATAGTTT

-903 CTACATTTCTGCCCGACTTATACTCCTCCAATGTGATTGTCCAATTCACC

-853 TCATATTTACTCATTTATTTATTTTACTATATTTCTCTCATTTATGACTT

-803 TTGTGGTAGAAAATTCAAAAAATATAACAAAACTCTAAAATAAATAAAAA

-753 GCCATGTAATTCATTTTTCTTTTTGGTACACCAGTTACTTTTTTTTTATT

-703 ACAATATAAGCATAAATTTAAAGAGTTATCTAAAAGTTTAACAAACATGA

-653 TGTTTATAAATAGTAATCATAAAAAACAATTCATTTTACGGTTTACAATT

-603 TAACATAAAAAGTAAATTTAACTTAAAAGAGTTTCTTGTCAGCTGAATAA

-553 GATAAAAGATTTTATAAGTTATTTTGGCAAGGAATTAAAGAAATTAAGTT

-503 CATCTTTATTCATGCATGTTTTTGTTAATTTTGTGTTATGTGACATAGAT

-453 TTTTAATAAAGATGAAGTTAAATTTTGTACTATGTAGAAATAACAGCACC

-403 AATCTGAAAAAAAAATAAAATTAATAGGACTGGGGATACTAAAGATAGTG

-353 GTTGAATAGTAAGTTAGTGGGATGATTAAGCTTTATATATAAAAAATAGG

-303 TTTAATTGCAATTTTAGTCAATCTATTTATACTGATTCATAAAATTGGTC

-253 CCC**TTATTT**TAAAAGTCCGACAATTTTAATCTATTTGTCTGATTTTTTAA

-203 TTAAAAAATGATGACATAAAATATTTTAAATAATATAATTTATAATAAGA

-153 TAATGTAAAATGATTAATAAATCTCATTTAATTTAAAATAATGTAAAATG

-103 AATTATCAACTACATCTCATTTAATAAAACAAATTTTTGAATTAGTTTAA

-53 TAAAATATAAACTCGTTTATCGACGTGATTGGAGATTGTCTTAGTCATTG

-3 GAC**A**TTGTCTTAGTAGCTAAGTCTTGTTCCCAAAATGGAGTGGAAAAATG

+48 ACTTTCCTTCCGTACCGGACTAAAAAAGCAGCTAAACACATTTGATATAA

+98 AGTATTGAAGTTGTGGACATCGACACCCATCTTAGTTAGTGAAGAAAATA

+148 ATG

***cis*-Element**	**Consensus**	**Motif position**
-300 Core/ Prolamin box	TGTAAAG	-1179
-300 element	TGHAAARK	-112, -150, 12331, -1489
AACA motif	AACAAAC	-664
ACGT element	ACGT	-31, -1519, -1597, -1962
Amylase box	TAACARA	-779, -1096, -1789
CAREs	CAACTC	-2167
DPBF Core	ACACNNG	-726
Pyrimidine box	CCTTTT	-2033, -2064, -2094
RY repeat	CATGCA	-493, -2208
SEF4 binding site	RTTTTTR	-71, -455, -486, -1080, -1137, -1999
Skn-1 motif	GTCAT	-10, -2073

S3 Fig. Promoter region of *CarF-box_PP2* showing seed specific *cis*-acting regulatory elements in colour. TSS (transcription start site) is depicted by bold larger font.
