# Supplementary material for: A New Set of ESTs from Chickpea (Cicer arietinum L.) Embryo Reveals Two Novel F-Box Genes, CarF-box_PP2 and CarF-box_LysM, with Potential Roles in Seed Development
Source: PLoS One. 2015 Mar 24;10(3):e0121100. doi: 10.1371/journal.pone.0121100 (PMC4372429; doi:10.1371/journal.pone.0121100)
Supplement: S4 Fig — The seed specific cis-acting regulatory elements are shown in colored blocks. Region underlined is the TATA box. TSS (transcription start site) is depicted by bold larger font. (PDF) [file pone.0121100.s008.pdf]

−614 GAAGGTGCAAAAGCTTGTTCATAAGCCCCTCATTTTATCTTATATGCAAT

−564 GTTGACAAATGATATAATCTTTACTGTGGTCCATTTATCTTGTAACTTAA

−514 TTATGCAATAGAAACAAAGACATTTTTTATAACTTGAAAGCCTCATCTAA

−464 AAGAGTAATGACAAAGGTATATACTAGATACAAAGTTTACATCAACTAAG

−414 ACTTCATTCAATTTATGAAGAGGGGAGCCAGTGAAACATCACTGGCATTG

−364 CCAATAGTTGAAGAGGGGAGCTATTTGGTTATCATGTATAAGTCTAAGAG

−314 AAAAGAGAAGGGAGGTTGTTTGTACAAAGGTTAAATGTTTGAACAATGAC

−264 TTCAAATTTTTATAAGCTTGGTCCTTTATCTCTATTTTTCTCTTTTTATC

−214 ATATCATCAGACAAATTACATTACTCTTTTTATCTCAAGATTTATAGGCT

−164 CAGACATCAATATTTTAATTATACGTAAAAAAAGACAAATTCTAATATTA

−114 CTATGTTTATCTTAGATAATTATTAATTCGACATCAATTAAATTACCCGG

−64 TCATCAAAAAAGAACCATGCAATTAATTG**TTATTT**CTAAAGTTTGGTGAG

−14 TAAATTAATAGAGA**C**CTTTACTAATGACTAAAATTGTTGAAAATATCCAT

+37 ATCCACATCCACGCGTATAAGATTTGTGGATAACTTATCGTTGGCAGGTG

+87 TAAGAAAGTAGTTCATCTTCCGAATG

| ***cis*-Element** | **Consensus** | **Motif position** |
|---|---|---|
| -300 element | TGHAAARK | -609 |
| ACGT element | ACGT | -143 |
| Amylase box | TAACARA | +30 |
| RY repeat | CATGCA | -49 |
| SEF4 binding site | RTTTTTR | -259 |
| Skn-1 motif | GTCAT | -65 |

**S4 Fig.** Promoter region of *CarF-box_LysM* showing seed specific *cis*-acting regulatory elements in colour. TSS (transcription start site) is depicted by bold larger font.
